# Precisely tailored shell thickness and Ln^3+^ content to produce multicolor emission from Nd^3+^-sensitized Gd^3+^-based core/shell/shell UCNPs through bi-directional energy transfer†

**DOI:** 10.1039/c9na00006b

**Published:** 2019-03-21

**Authors:** York E. Serge Correales, Chanchal Hazra, Sajjad Ullah, Laís R. Lima, Sidney J. L. Ribeiro

**Affiliations:** Institute of Chemistry, São Paulo State University, UNESP 14800-060 Araraquara SP Brazil sidney.jl.ribeiro@unesp.br sjlribeiro@gmail.com chanchalhazra007@gmail.com; Institute of Chemical Sciences, University of Peshawar 25120 Peshawar Pakistan

## Abstract

Lanthanide (Ln^3+^)-doped upconversion nanoparticles (UCNPs) have been paid great attention as multiplexing agents due to their numerous uses in biological and clinical applications such as bioimaging and magnetic resonance imaging (MRI), to name a few. To achieve efficient multicolor emission from UCNPs under single 808 nm excitation and avoid detrimental cross-relaxations between the Ln^3+^ activator ions (positioned in either the core and/or shell in the core/shell), it is essential to design an adequate nanoparticle architecture. Herein, we demonstrate the tailoring of multicolor upconversion luminescence (UCL) from Nd^3+^-sensitized Gd^3+^-based core/shell/shell UCNPs with an architecture represented as NaGdF_4_:Tm^3+^(0.75)/Yb^3+^(40)/Ca^2+^(7)/Nd^3+^(1)@NaGdF_4_:Ca^2+^(7)/Nd^3+^(30)@NaGdF_4_:Yb^3+^(40)/Ca^2+^(7)/Nd^3+^(1)/Er^3+^(*X* = 1, 2, 3, 5, 7) [hereafter named CSS (Er^3+^ = 1, 2, 3, 5 and 7 mol%)]. Such UCNPs can be excited at a single wavelength (∼808 nm) without generation of any local heat. Incorporation of substantial Nd^3+^-sensitizers with an appropriate concentration in the middle layer allows efficient harvesting of excitation light which migrates bi-directionally across the core/shell interfaces in sync to produce blue emission from Tm^3+^ (activator) ions in the core as well as green and red emission from Er^3+^ (activator) ions in the outermost shell. Introduction of Ca^2+^ lowers the local crystal field symmetry around Ln^3+^ ions and subsequently affects their intra 4f–4f transition probability, thus enhancing the upconversion efficiency of the UCNPs. By simple and precise control of the shell thickness along with tuning the content of Ln^3+^ ions in each domain, multicolor UCL can be produced, ranging from blue to white. We envision that our sub-20 nm sized Nd^3+^-sensitized Gd^3+^-based UCNPs are not only potential candidates for a variety of multiplexed biological applications (without impediment of any heating effect), but also can act as MRI contrast agents in clinical diagnosis.

## Abstract 2

Precise tailoring of the shell thickness and Ln^3+^ content produced multicolour emissions from core/shell/shell UCNPs *via* a bi-directional energy transfer mechanism.

## Graphical abstract

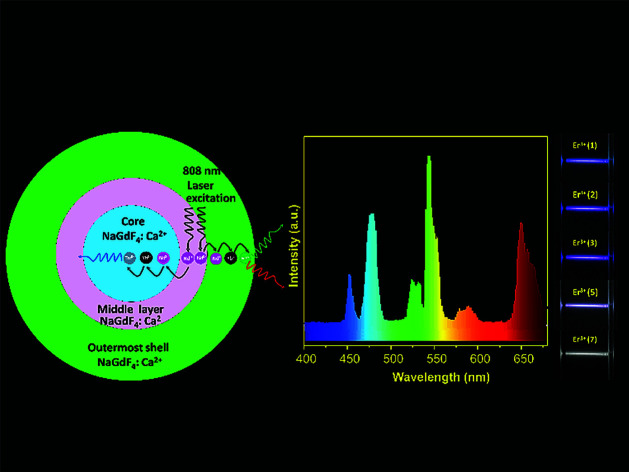

## Introduction

In view of the recent research upsurge, multicolor luminescence from photoluminescent probes is of great interest due to its widespread use in chemical and biological sciences.^1–7^ The multicolor luminescence provides remarkable insight into molecular biological processes, and, using a number of color-encoded probes, makes a large number of biochemical entities feasible for high-throughput sequencing technologies together with brief sequencing chemistry.^8–10^ Along with multicolor luminescence, multiplexing experiments are adequately used in human genome decoding, and biological and clinical applications.^11–13^ To date, there are several reports available on photoluminescent probes that have been used as multiplexing agents in bio-system analysis and therapy applications.^14–18^ Among them, Ln^3+^-doped UCNPs hold promise because of their unique anti-Stokes (known as upconversion) optical properties.^19–24^ Typically, these Ln^3+^-doped nanoparticles are composed of sensitizers (*e.g.*, Yb^3+^/Nd^3+^) and activators (Er^3+^/Tm^3+^/Ho^3+^) which are spatially distributed in an appropriate host matrix or in a dilute host–guest system. Successive absorption of near infrared (NIR) photons and/or energy transfer (ET) generally promote the activators to higher excited states and concurrently lead to the emission of higher energy radiation spanning over a wide region, from the ultraviolet (UV) to NIR spectral regions.^25–29^ The relatively high penetration depth of NIR light in biological structures causes a minimal autofluorescence background signal and low biotoxicity, which again make UCNPs ideal candidates for a wide range of biological applications such as sensing, imaging and therapy, among others.^30–38^ Moreover, compact, inexpensive continuous-wave NIR diode lasers can be used to excite UCNPs, leading to the generation of multiphoton UCL which is an important additive to conventional multiphoton microscopy. This characteristic physiochemical property of UCNPs also allows their extensive use in biological domains.^39–41^

Doping different types of activators (*e.g.*, Er^3+^/Tm^3+^/Ho^3+^) together with sensitizers (*e.g.*, Yb^3+^) with a precise concentration in a suitable host matrix can produce multicolor UCL under single NIR wavelength excitation at 980 nm, which corresponds to the absorption of Yb^3+^ ions.^42,43^ However, this strategy can lead to cross-relaxation processes between the different types of activators, resulting in lower UCL efficiency from the UCNPs.^44–46^ Moreover, in the Yb^3+^-sensitized upconversion process, the excitation wavelength (980 nm) is strongly absorbed by water molecules in biological environment which causes local heating problems in the vicinity of cells and tissues, thus damaging them.^42,47^ To address this problem, recent research has been focused on the extension of the upconversion excitation spectrum to shorter wavelengths (especially, in the vicinity of 808 nm), where water molecules do not absorb significantly.^48–52^ Nevertheless, efficient multicolor UCL output from Nd^3+^-sensitized UCNPs remains vague until this point.

Furthermore, the multicolor UCL from Nd^3+^-sensitized UCNPs, obtained through a combination of different types of activators (such as Er^3+^ and Tm^3+^) in a single domain, can introduce deleterious cross-relaxation quenching processes, leading to low UCL efficiency from UCNPs. Therefore, it is necessary to separate different types of activators at a nanoscale distance. Moreover, to date, Nd^3+^-sensitized core/shell UCNPs have typically been exploited to transfer the energy from a Nd^3+^-doped light-harvesting layer across the core/shell interface to a Yb^3+^/X^3+^ (X = Er, Ho or Tm) co-doped layer in one single direction. This uni-directional ET process provides no plausible scope to sensitize two different types of activators in separated layers which again leads to the decrease in UCL efficiency, knowing that the efficiency depends on the distance between the Nd^3+^-doped layer and the activator-doped layer. It is worth mentioning that the efficiency of Nd^3+^ → Yb^3+^ non-radiative ET is directly proportional to the number of Yb^3+^ ions per Nd^3+^ ion in the doped region. In fact, the ability to harvest light most efficiently by UCNPs at 808 nm is also directed by the total number of doped Nd^3+^ ions. Thus, to achieve efficient multicolor UCL, it is essential to optimize the concentration of Yb^3+^ ions and Nd^3+^ ions in their defined domains. Recently, Hao *et al.* reported that the Tm^3+^ and Er^3+^ ions doped into sandwich-structured UCNPs of core/shell/shell NaYbF_4_:Tm^3+^@CaF_2_:Nd^3+^@CaF_2_:Er^3+^ effectively produce multicolour UCL *via* a bi-directional ET process.^53^ However, to our knowledge, there is no report available on the use of a bi-directional ET process along with precise control of the shell thickness and proper tuning of the content of Ln^3+^ ions in each domain to produce bright multicolour emission from Nd^3+^-sensitized Gd^3+^-based core/shell/shell UCNPs. Such strategies could be a potential tool for bio-imaging and MRI dual modal applications in the near future.

In this context, we report Nd^3+^-sensitized Gd^3+^-based core/shell/shell (CSS) UCNPs to achieve multicolor UCL under single wavelength excitation at 808 nm. In particular, NaGdF_4_ has been selected as a host matrix (both as the core and the shell layer) as it allows the formation of small sized nanoparticles. In small sized UCNPs, the surface-to-volume ratio is very high and a large portion of dopant ions are located at the surface. Moreover, with proper functionalization, Gd^3+^-based core/shell/shell UCNPs could effectively serve as MRI contrast agents in medical diagnosis. Ca^2+^ was chosen as it has lower valence than Gd^3+^ ions. Upon replacement of some Gd^3+^ ions by Ca^2+^ ions, a charge imbalance in NaGdF_4_ occurs which lowers the local crystal field symmetry around Yb^3+^, Er^3+^ and Tm^3+^ ions and subsequently affects the intra 4f–4f transition probability of the Ln^3+^ ions, thus enhancing the upconversion efficiency.^34,54–56^ The Nd^3+^ sensitizer is doped at a high concentration (30 mol%) in the middle layer to effectively and efficiently absorb the excitation light in this region and simultaneously conveys it to core and outermost shell in a bi-directional manner to emit efficient multicolor emissions from the nanoparticles. Tm^3+^ and Er^3+^ activators are separated into the inner core and the outermost shell, respectively, to prevent deleterious cross-relaxation processes between them. Moreover, the core/shell/shell nanoconfiguration allows simultaneous non-radiative ET from the middle layer to both the Tm^3+^-doped core and Er^3+^-doped outermost shell. Since high concentrations of Yb^3+^ ions (40 mol%) are incorporated in both the outermost shell and core domain, efficient core/shell interface energy migration takes place from the Nd^3+^ ions to the Yb^3+^ ions, which then sensitizes the activator ions (both Er^3+^ and Tm^3+^) to produce bright UCL. A lower doping of Nd^3+^ (1 mol%) in the outermost shell and in the core region is expected to assist the energy migration across the core/shell interfaces through a resonant ET process between Nd^3+^ ions. We demonstrated that the CSS (Er^3+^ = 1, 2, 3, 5 and 7 mol%) UCNPs are able to emit efficient multicolor UCL, ranging from blue to white under laser excitation at 808 nm. Tailoring the shell thickness, along with a precise control of the concentration of sensitizers and activators in their defined domains, allows us to achieve such tunable multicolor UCL from our designed sub-20 nm sized core/shell/shell UCNPs.

## Experimental

### Materials

Gd_2_O_3_ (99.99%, Sigma-Aldrich), Yb_2_O_3_ (99.99%, Sigma-Aldrich), Er_2_O_3_ (99.99%, Sigma-Aldrich), Nd_2_O_3_ (99.99%, Sigma-Aldrich), Tm_2_O_3_ (99.99%, Sigma-Aldrich), CaCl_2_ (Synth), NaOH (Synth), NH_4_F (Merck), methanol (CH_3_OH, Synth), 1-octadecene (ODE, Aldrich), oleic acid (OA, Aldrich), cyclohexane (Synth, Brazil), and ethanol (C_2_H_5_OH, Synth, Brazil) were used in this work. All chemicals were used without further purification.

### Methods

#### Synthesis of core NaGdF_4_:Tm^3+^(0.75)/Yb^3+^(40)/Nd^3+^(1)/Ca^2+^(7)

The core NaGdF_4_:Tm^3+^(0.75)/Yb^3+^(40)/Nd^3+^(1)/Ca^2+^(7) UCNPs were synthesized following a modified co-precipitation method according to a literature report.^57^ In a typical experiment, 4 mL aqueous solution (0.2 M) of LnCl_3_·6H_2_O (Ln = Gd, Yb, Nd and Tm) and CaCl_2_ were added to a 100 mL three-necked flask containing oleic acid (6 mL) and 1-octadecene (14 mL). The resulting mixture was heated to 150 °C and kept for 1 h to form lanthanide oleate complexes and then cooled down to room temperature. Subsequently, a methanol solution (10 mL) containing NH_4_F (3.25 mmol) and NaOH (2.0 mmol) was added and the mixture was stirred at 50 °C for 30 min. The temperature of the reaction mixture was then increased to 110 °C to remove methanol. Upon removal of methanol and keeping the reaction mixture at 110 °C under vacuum for 15 min, the solution was heated to 300 °C under a N_2_ atmosphere and kept for 30 min before cooling down to room temperature naturally. The resulting nanoparticles were precipitated out by the addition of ethanol, collected by centrifugation, washed with cyclohexane, ethanol and methanol and finally redispersed in 4 mL cyclohexane.

#### Synthesis of core/shell NaGdF_4_:Tm^3+^(0.75)/Yb^3+^(40)/Nd^3+^(1)/Ca^2+^(7)@NaGdF_4_:Ca^2+^(7)/Nd^3+^(*X*) (*X* = 5, 10, 20, 30 and 50 mol%) [hereafter named CS (Nd^3+^ = 5, 10, 20, 30 and 50 mol%)]

In a typical experiment, a 4 mL solution (0.2 M) of LnCl_3_.6H_2_O (Ln = Gd, Nd) and CaCl_2_ in water was added to a 100 mL three-necked flask containing oleic acid (6 mL) and 1-octadecene (14 mL). The mixture was then heated to 150 °C and kept for 1 h with magnetic stirring and then cooled down to 50 °C naturally. NaGdF_4_:Tm^3+^(0.75)/Yb^3+^(40)/Nd^3+^(1)/Ca^2+^(7) core UCNPs in 4 mL cyclohexane were then added along with 10 mL methanol solution of NH_4_F (3.25 mmol) and NaOH (2.0 mmol). The resulting mixture was stirred at 50 °C for 30 min. The temperature of the reaction mixture was increased to 110 °C to remove the methanol. Upon removal of the methanol and keeping the reaction mixture at 110 °C under vacuum for 15 min, the solution was then heated to 300 °C under a N_2_ atmosphere and kept for 45 min before cooling down to room temperature naturally. The resulting nanoparticles were precipitated out by the addition of ethanol, collected by centrifugation, washed with cyclohexane, ethanol, and methanol and finally redispersed in 4 mL cyclohexane.

#### Synthesis of core/shell/shell [CSS (Er^3+^ = 1, 2, 3, 5 and 7 mol%)]

In a typical experiment, a 4 mL solution (0.2 M) of LnCl_3_.6H_2_O [Ln = Gd, Nd, Yb, Er (*X* = 1,2,3,5,7)] and CaCl_2_ in water was added to a 100 mL three-necked flask containing oleic acid (6 mL) and 1-octadecene (14 mL). The mixture was then heated to 150 °C and kept for 1 h with magnetic stirring and then cooled down to 50 °C naturally. The CS (Nd^3+^ = 30 mol%) UCNPs in 4 mL cyclohexane were then added along with a 10 mL methanol solution of NH_4_F (3.25 mmol) and NaOH (2.0 mmol). The resulting mixture was stirred at 50 °C for 30 min. The temperature of the reaction mixture was increased to 110 °C to remove the methanol. Upon removal of the methanol and keeping the reaction mixture at 110 °C under vacuum for 15 min, the solution was then heated to 300 °C under a N_2_ atmosphere and kept for 1 h before cooling down to room temperature naturally. The resulting nanoparticles were precipitated out by the addition of ethanol, collected by centrifugation, washed with cyclohexane, ethanol, and methanol and finally redispersed in 4 mL cyclohexane. Different shell thickness controlled CS (Nd^3+^ = 30 mol%) and CSS (Er^3+^ = 5 mol%) UCNPs were synthesized following identical procedures to those mentioned above, except for a change in the amount of shell precursors and methanolic solution of NH_4_F and NaOH.

### Characterization

#### Structure and morphology

Fourier transform infrared (FTIR) spectra were collected using a PerkinElmer Spectrum 1000 FTIR spectrometer with a resolution of 2 cm^−1^ and averaged over four scans. Room temperature optical absorption spectra of the samples were recorded on a Varian model Cary 5000 spectrophotometer. The samples were taken in a 3 mL quartz cuvette (path length, 2 cm). The XRD measurements were performed on a powder X-ray diffractometer (Rigaku-RINT 2000) using Ni-filtered Cu Kα X-ray radiation (1.540 Å) from a rotating Cu anode operated at 30 mA and 30 kV. The diffracted X-rays in the 2-theta range of 10–60° were monitored with a linear D/Tex detector at a scan rate of 1° min^−1^. TEM analyses of the samples supported on holey carbon-coated copper grids were carried out using an FEI TECNAI (G2 F20) transmission electron microscope operated at an accelerating voltage of 200 kV. Before the imaging, each sample was further diluted in cyclohexane and sonicated for 1 min. One drop of the dispersion was deposited on a carbon-coated copper grid and the solvent was allowed to slowly evaporate.

#### Optical properties

Upconversion emission spectra of the core, core/shell and core/shell/shell UCNPs were acquired using a Horiba Jobin Yvon spectrofluorometer (model fluorolog-3 FL3-122) equipped with a photomultiplier tube (model R 928 P, Spex) sensitive between 250 and 850 nm. An 808 nm laser diode (from DMC, Brazil) coupled with an optical fiber (200 μm diameter) was utilized as the excitation source. All spectra were acquired in identical fashion under the same experimental conditions. The laser power used in all measurements was 7 W cm^−2^, determined using a Newport power meter, model 2935-C.

## Results and discussion

### Structural characterization of materials

**Scheme 1:**
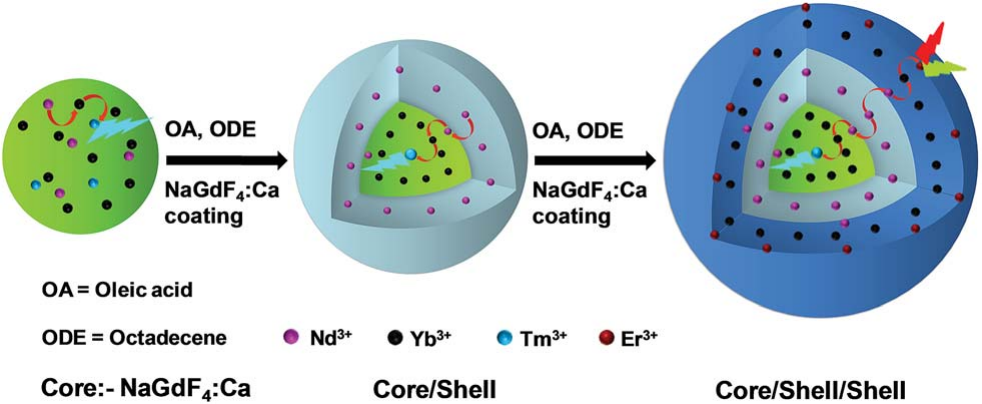
Schematic illustration of the synthesis of CSS UCNPs following a seed-mediated layer-by-layer growth method.

**Fig. 1:**
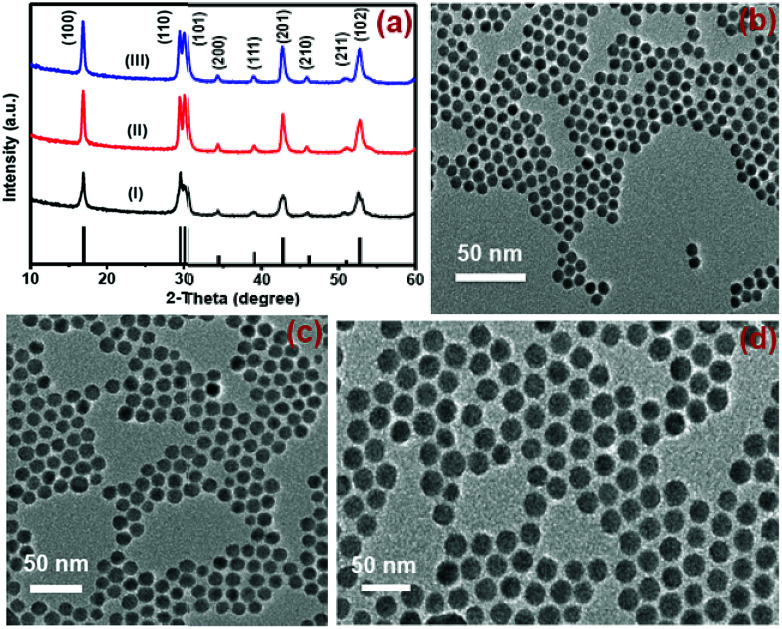
(a) XRD patterns of the NaGdF_4_:Tm^3+^(0.75)/Yb^3+^(40)/Nd^3+^(1)/Ca^2+^(7) core (i), CS (30 mol% Nd^3+^) (ii) and CSS (5 mol% Er^3+^) (iii) UCNPs. (b, c, and d) present the TEM images of these core, CS and CSS UCNPs, respectively.

Nd^3+^-sensitized Gd^3+^-based CSS UCNPs were successfully synthesized following a modified seed-mediated layer-by-layer growth method according to a literature report^57^ (Scheme 1). Phase analysis of the core, CS (30 mol% Nd^3+^) and CSS (5 mol% Er^3+^) UCNPs was carried out using powder X-ray diffraction (XRD) measurements (Fig. 1a). All diffraction peaks perfectly match with the standard diffraction pattern of hexagonal NaGdF_4_ crystals (JCPDS no: 27-0699). The shape and size of the nanoparticles were examined by TEM. TEM images indicate the formation of uniform and monodisperse nanoparticles with an average diameter of 7 nm, 12 nm and 19 nm for core (NaGdF_4_:Tm^3+^(0.75)/Yb^3+^(40)/Nd^3+^(1)/Ca^2+^(7)), CS (30 mol% Nd^3+^) and CSS (5 mol% Er^3+^) UCNPs, respectively (Fig. 1b, c and d). The shell thickness of the CS and CSS UCNPs is thus estimated to be around 2.5 and 3.5 nm, respectively. Monodispersity in the size of these core, CS and CSS UCNPs is evident from the size distribution histograms shown in Fig. S1.† Along with the size increase (core → CS → CSS), there is hardly any change in the morphology observed, indicating the homogeneous nature of shell growth over core and CS UCNPs. The high resolution TEM results further supported the formation of high quality hexagonal phase core, CS and CSS UCNPs (Fig. S2†). The qualitative chemical composition of these UCNPs was studied by energy-dispersive X-ray spectroscopy (EDS) (Fig. S3†). The EDS spectra of the samples show the presence of Gd, Nd, Ca, Yb, and Tm, Gd, Nd, Ca, Yb, and Tm and Gd, Nd, Ca, Yb, Tm, and Er for core, CS and CSS UCNPs, respectively, implying the layer-by-layer growth of shells on core nanoparticles. The capping of core, CS and CSS UCNPs with oleic acid onto the surface of the same is confirmed by the appearance of strong carbonyl stretching vibration near 1500 cm^−1^ (Fig. S4†). This frequency is much lower than that observed for the free oleic acid molecules (1700 cm^−1^), indicating strong binding of the ligands (oleic acid) onto the surface of the nanoparticles.

### Study of optical properties

**Fig. 2:**
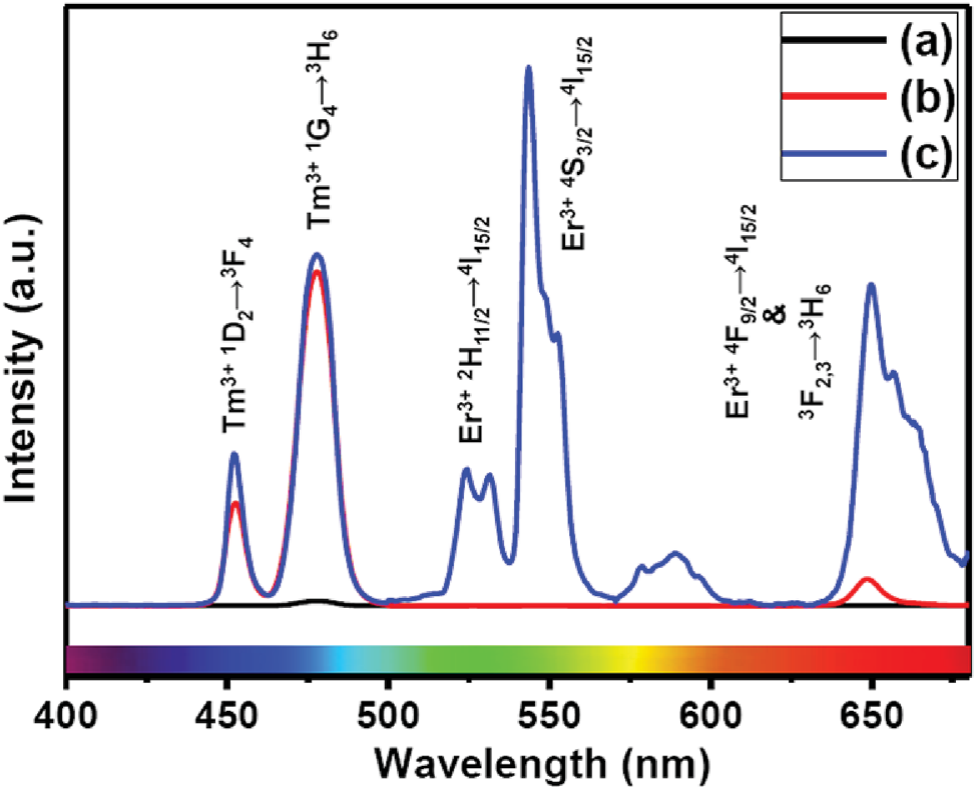
UCL spectra of the (a) core NaGdF_4_:Tm^3+^(0.75)/Yb^3+^(40)/Nd^3+^(1)/Ca^2+^(7), (b) CS (30 mol% Nd^3+^) and (c) CSS (5 mol% Er^3+^) UCNPs.

The presence of Yb^3+^ ions (along with 1 mol% Nd^3+^ ions) in core, (Yb^3+^ + Nd^3+^) ions in CS (30 mol% Nd^3+^) and (Yb^3+^ + Nd^3+^) ions in CSS (5 mol% Er^3+^) UCNPs is confirmed by the presence of their characteristic peaks in the electronic absorption spectra of the samples (Fig. S5†). In comparison to core UCNPs, the absorption intensity of the characteristic peaks of Nd^3+^ ions increases with increase in the Nd^3+^ concentration in the CS and CSS UCNPs, suggesting efficient incorporation of Nd^3+^ ions in the shell of CS and in the middle layer of CSS UCNPs. To justify the importance of a Nd^3+^-enriched shell to efficiently harvest light at 808 nm for simultaneous sensitization of both Tm^3+^ and Er^3+^ activators to produce bright multicolor UCL, we first prepared the core-NaGdF_4_:Tm^3+^(0.75)/Yb^3+^(40)/Nd^3+^(1)/Ca^2+^(7), CS (30 mol% Nd^3+^) and CSS (5 mol% Er^3+^) UCNPs. Fig. 2 shows the upconversion emission spectra for these core and CS UCNPs obtained using 808 nm laser excitation. The UCL peaks centered around 451 and 477 nm are assigned to the ^1^D_2_ → ^3^F_4_ and ^1^G_4_ → ^3^H_6_ transitions of Tm^3+^ ions, respectively. The calculated integrated UCL intensity of the blue (442–505 nm) emission of CS UCNPs is 77 times higher than that of core UCNPs. The reason behind this observation might be explained on the basis of two factors. First, surface related harsh quenching of the core NaGdF_4_:Tm^3+^(0.75)/Yb^3+^(40)/Nd^3+^(1)/Ca^2+^(7) is diminished by coating the NaGdF_4_:Nd^3+^(30)/Ca^2+^(7) shell over the former. Secondly, the high concentration of Nd^3+^ ions (30 mol%) in the shell of the core/shell UCNPs might harvest the light at 808 nm more efficiently and subsequently transfer it to the core region to sensitize Yb^3+^ ions and simultaneously the Tm^3+^ ions (activator) to produce blue emission.

**Fig. 3:**
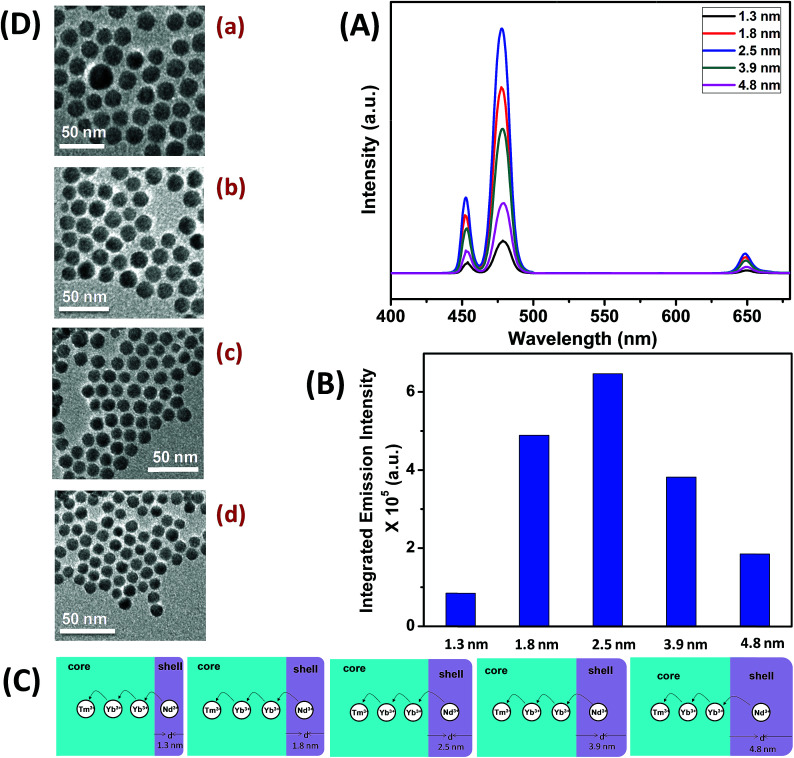
(A) UCL spectra of CS (30 mol% Nd^3+^) UCNPs with different thickness of the NaGdF_4_:Ca^2+^(7)/Nd^3+^(30) shell (*d* = 1.3–4.8 nm). (B) Integrated intensity of the blue (462–505 nm) emission of CS (30 mol% Nd^3+^) UCNPs with different shell thickness (*d* = 1.3–4.8 nm). (C) Mechanistic investigation of interfacial ET from the Nd^3+^-containing shell to the Tm^3+^ containing core in different shell thickness controlled (*d* = 1.3–4.8 nm) CS (30 mol% Nd^3+^) UCNPs. (D) TEM images of CS (30 mol% Nd^3+^) UCNPs with different shell thickness ((a) 1.3 nm, (b) 1.8 nm, (c) 3.9 nm and (d) 4.8 nm).

To understand the effect of shell thickness on the UCL intensity from CS (30 mol% Nd^3+^), the core particles were coated with a shell of variable thickness (*i.e.*, 1.3, 1.8, 2.5, 3.9 and 4.8 nm) using epitaxial growth (Fig. 3C and D). As shown in Fig. 3A, the luminescence intensity first increases, reaching a maximum for a shell thickness of 2.5 nm, and then decreases. From Fig. 3B, it could be noted that the calculated integrated intensity of the blue (462–505 nm) emission of CS UCNPs with 2.5 nm shell thickness is 7.6 and 3.5 times higher than that of CS nanoparticles with 1.3 and 4.8 nm shell thickness, respectively. This result clearly suggests that shell thickness plays a pivotal role in the UCL intensity of CS UCNPs. Thinner shells (*i.e.*, shell thickness < 2.5 nm) allow the activator (Tm^3+^, 0.75 mol% in the core) and the sensitizer (Nd^3+^, 30 mol% in the shell) to come closer in proximity. Hence, a good overlap between emission of Tm^3+^ ions and the absorption of Nd^3+^ ions could be expected which assures an efficient back ET from Tm^3+^ ions to Nd^3+^ ions, leading to a decrease in blue UCL from the CS UCNPs. On the other hand, with an increase in the shell thickness (>2.5 nm), though the shell with 30 mol% Nd^3+^ efficiently harvests excitation light, it does not allow it to migrate effectively to Yb^3+^ ions (in core) across the core/shell interfaces to produce efficient blue emission from Tm^3+^ (activator) ions in the core. In fact, such CS UCNPs with a thicker shell (>2.5 nm) enable excitation energy to travel a long Nd^3+^–Yb^3+^ distance, which again leads to the decrease in the UCL intensity from the nanoparticles.

Upon growth of the second shell (*i.e.*, NaGdF_4_:Yb^3+^(40)/Nd^3+^(1)/Ca^2+^(7)/Er^3+^(5) onto the surface of CS (30 mol% Nd^3+^) UCNPs, the UCL from both activators (Er^3+^ and Tm^3+^) was observed together (Fig. 2, spectrum c). Along with the ^1^D_2_ → ^3^F_4_ and ^1^G_4_ → ^3^H_6_ transitions of Tm^3+^ ions, the green emission peak centered at 544 nm (^4^S_3/2_ → ^4^I_15/2_) and the red emission peak centered at 650 nm (^4^F_9/2_ → ^4^I_15/2_) from Er^3+^ ions are also observed. This result substantiates that the Nd^3+^ enriched shell can harvest excitation light more efficiently and allows migration of energy across the core/shell interface to both Tm^3+^ ions in the core and Er^3+^ in the outermost shell to produce multicolor UCL from CSS UCNPs.

**Fig. 4:**
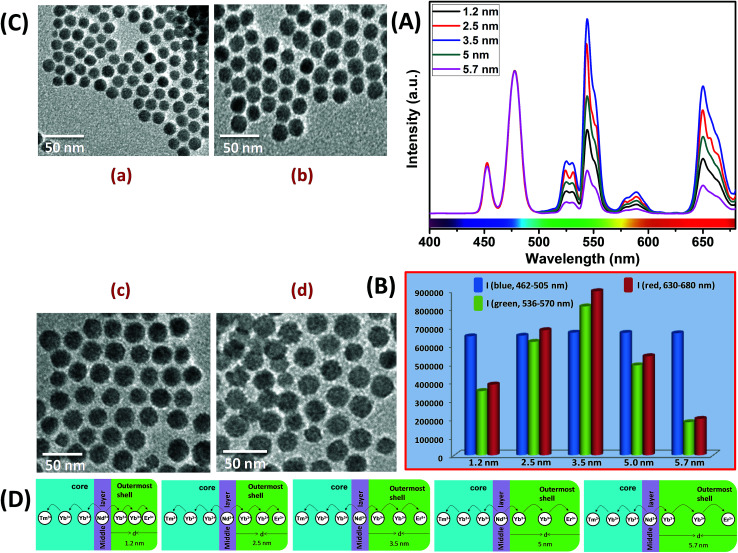
(A) UCL spectra of CSS UCNPs (5 mol% Er^3+^) with different thickness of the outermost shell over CS (30 mol% Nd^3+^) UCNPs (*d* = 1.2–5.7 nm). (B) Integrated intensity of blue (Tm^3+^, 462–505 nm), green (Er^3+^, 536–570 nm) and red (Er^3+^, 630–680 nm) emissions of CSS UCNPs with different outermost shell thickness (*d* = 1.2–5.7 nm). Note that, the UCL spectra and integrated emissions in (A) and (B), respectively, are normalized to the blue (462–505 nm) emission. (C) TEM images of CSS (5 mol% Er^3+^) UCNPs with different outermost shell thickness ((a) 1.2 nm, (b) 2.5 nm, (c) 5.0 nm and (d) 5.7 nm). (D) Mechanistic investigation of interfacial ET from the Nd^3+^-containing middle shell layer to the Tm^3+^ containing core (with defined shell thickness *d* = 2.5 nm) and Er^3+^ containing outermost shell (with different shell thickness *d* = 1.2–5.7 nm) in CSS (5 mol% Er^3+^) UCNPs.

Similar to CS (30 mol% Nd^3+^), to examine the effect of shell thickness on the UCL intensity from CSS (5 mol% Er^3+^), CS (30 mol% Nd^3+^) was used as a seed for the epitaxial growth of outermost shells with different thickness (1.2, 2.5, 3.5, 5 and 5.7 nm) (Fig. 4C and D). From Fig. 4A, a shell thickness of 3.5 nm results in the highest UCL. From Fig. 4B, it can be clearly observed that the calculated integrated intensity of the green (536–570 nm) emission of these CSS UCNPs with 3.5 nm shell thickness is 2.3 and 4.5 times higher than that of CSS nanoparticles with 1.2 and 5.7 nm outermost shell thicknesses, respectively. A similar trend was observed for red (630–680 nm) emission too. Thinner outermost shells (*i.e.*, shell thickness 1.2 and 2.5 nm) allow the activator (Er^3+^, 5 mol% in the outermost shell) and the sensitizer (Nd^3+^, 30 mol% in the middle shell layer) to come closer in proximity. Therefore, in this case too, a good overlap between emission of Er^3+^ ions and the absorption of Nd^3+^ ions can be expected which manifests itself in the form of an efficient back ET from Er^3+^ ions to Nd^3+^ ions, leading to the decrease in UCL (green + red) from the CSS UCNPs. On the other hand, with an increase in the outermost shell thickness (>3.5 nm), the shell with 30 mol% Nd^3+^, despite efficiently harvesting excitation light, does not allow it to migrate efficiently to Yb^3+^ ions (40 mol%, outermost shell) concurrently to produce green and red emissions from Er^3+^ (activator) ions in the outermost shell.

Moreover, like the CS UCNPs, the CSS UCNPs with a thicker shell (>3.5 nm) make the excitation energy travel a long Nd^3+^–Yb^3+^ distance, which again leads to a decrease in the green and red UCL intensity from the nanoparticles. The UCL spectra and integrated emissions in Fig. 4A and B, respectively, are normalized to the blue (462–505 nm) emission. Actually, to prepare CSS nanoparticles *via* the epitaxial growth mechanism, we used CS nanoparticles with similar size in each reaction. We only varied the amount of outermost shell precursor to deposit an outermost shell (with different thickness) over CS nanoparticles. All other synthesis parameters (time, temperature *etc.*) were kept constant. Hence, there was no remarkable change in the blue emission intensity as the energy transfer efficiency of Nd → Yb → Tm remained unaltered in each case. Though we acquired all the spectra under identical experimental conditions, still we observed a slight change in the blue emission. To further clarify the effect of outermost shell thickness on the green and red emission of Er^3+^ ions (doped in outermost shell), we normalized the whole spectra and intensity with respect to the blue emission. The original spectra and intensity (without normalization) are shown in Fig. S6 and Table S1,† respectively.

**Fig. 5:**
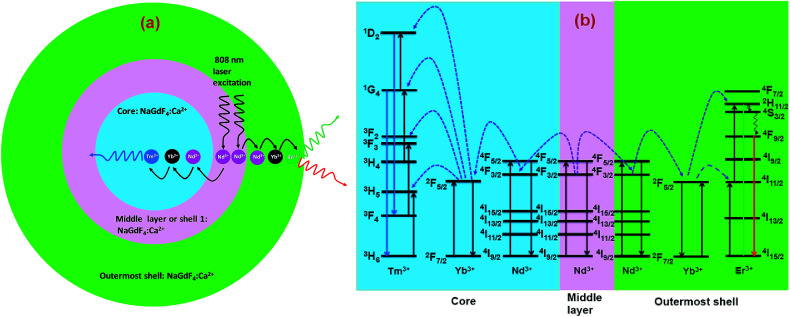
(a) Schematic illustration of the non-radiative ET mechanism of Tm^3+^ ← Yb^3+^ ← Nd^3+^ → Yb^3+^ → Er^3+^ in CSS (5 mol% Er^3+^) UCNPs (808 nm laser excitation). (b) Schematic illustration of ET pathways to produce bright UCL from both the core (blue, Tm^3+^) and the outermost shell (red and green, Er^3+^) in the same CSS UCNPs upon 808 nm excitation.

**Fig. 6:**
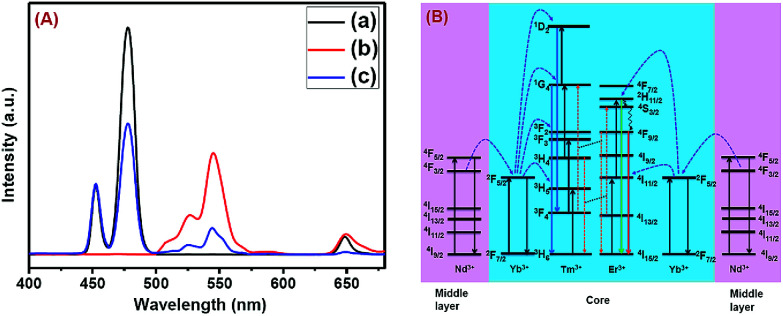
(A) Comparison of UCL spectra of (a) NaGdF_4_:Tm^3+^(0.75)/Yb^3+^(40)/Nd^3+^(1)/Ca^2+^(7)@NaGdF_4_:Ca^2+^(7)/Nd^3+^(30), (b) NaGdF_4_:Er^3+^(2)/Yb^3+^(40)/Nd^3+^(1)/Ca^2+^(7)@NaGdF_4_:Ca^2+^(7)/Nd^3+^(30) and (c) NaGdF_4_:Tm^3+^(0.75)/Er^3+^(2)/Yb^3+^(40)/Nd^3+^(1)/Ca^2+^(7)@NaGdF_4_:Ca^2+^(7)/Nd^3+^(30) core/shell UCNPs, upon 808 nm laser excitation, to verify the cross-relaxation between Tm^3+^ and Er^3+^ ions. (B) Energy level diagram indicating the cross-relaxation mechanism between Tm^3+^ and Er^3+^ ions in NaGdF_4_:Tm^3+^(0.75)/Er^3+^(2)/Yb^3+^(40)/Nd^3+^(1)/Ca^2+^(7)@NaGdF_4_:Ca^2+^(7)/Nd^3+^(30) core/shell UCNPs, upon 808 nm laser excitation.

The CSS (5 mol% Er^3+^) nanostructure thus plays an important role in separating two activators (Tm^3+^ and Er^3+^) to mitigate deleterious cross-relaxation processes between them. Fig. 5a depicts a schematic route of nonradiative ET processes of Tm^3+^ ← Yb^3+^ ← Nd^3+^ → Yb^3+^ → Er^3+^ in the CSS UCNPs, upon 808 nm excitation. The schematic illustration of ET pathways to produce bright UCL from both the core and the outermost shell in the designed CSS UCNPs (upon 808 nm excitation) is shown in Fig. 5b. Moreover, to better understand, we recorded the UCL spectra of three different NaGdF_4_:Tm^3+^(0.75)/Yb^3+^(40)/Nd^3+^(1)/Ca^2+^(7)@NaGdF_4_:Ca^2+^(7)/Nd^3+^(30), NaGdF_4_:Er^3+^(2)/Yb^3+^(40)/Nd^3+^(1)/Ca^2+^(7)@NaGdF_4_:Ca^2+^(7)/Nd^3+^(30) and NaGdF_4_:Tm^3+^(0.75)/Er^3+^(2)/Yb^3+^(40)/Nd^3+^(1)/Ca^2+^(7)@NaGdF_4_:Ca^2+^(7)/Nd^3+^(30) core/shell UCNPs to verify the adverse cross-relaxation processes between the activator ions (Tm^3+^ and Er^3+^). From Fig. 6A, it is clearly observed that the UCL intensity is significantly reduced when Tm^3+^ and Er^3+^ ions are doped together in the core region (spectrum c), indicating the unwanted cross-relaxation process between them. As a reasonable explanation of our observation, a simplified energy level diagram depicting the cross-relaxation quenching mechanisms between Tm^3+^ and Er^3+^ (under 808 nm excitation) in Nd^3+^-sensitized Gd^3+^-based core/shell UCNPs is shown in Fig. 6B. In order to achieve efficient multicolor UCL from CSS (5 mol% Er^3+^) UCNPs, it is essential to first optimize the concentration of Tm^3+^ ions in the core region of CS (30 mol% Nd^3+^) nanoparticles. To achieve the utmost blue emission (808 nm excitation), the optimal concentration of Tm^3+^ ions in CS nanoparticles (against 40 mol% Yb^3+^ ions) was found to be 0.75 mol% (Fig. S7†). The reason behind the use of such high mol% of Yb^3+^ ions has also been explained in this article (*vide infra*). We also investigated the effect of Nd^3+^ concentration in the middle layer of CS UCNPs and the optimum concentration of Nd^3+^ ion was determined to be 30% (Fig. S8†). The gradual increase of Nd^3+^ concentration in the middle layer is consistent with the overall increase in absorption of excitation energy, leading to the utmost photon harvesting efficiency and, concurrently, the strongest UCL intensity from the UCNPs. However, at a Nd^3+^ concentration higher than 30 mol%, the mutual interaction between Nd^3+^ ions increases in the shell. This enhanced interaction consumes the excitation energy, leading to less efficient energy migration across the core/shell interface to the Tm^3+^-doped core. We believe that a similar phenomenon might occur in the Er^3+^-doped outermost shell in CSS UCNPs. Thus, multicolor UCL intensity from the UCNPs is greatly reduced. Moreover, a good overlap between the emission of Tm^3+^ and Er^3+^ ions with the absorption of Nd^3+^ ions ensures an efficient back ET from Tm^3+^ or Er^3+^ ions to Nd^3+^ ions which again leads to a decrease in UCL from the nanoparticles. Upon variation in the concentration of Nd^3+^ ions in the shell, the crystalline phase and size of the CS UCNPs remain intact (Fig. S9†). It is worth mentioning that the coating of at least one inert shell over CS UCNPs is necessary to preserve excitation energy and simultaneously achieve strong luminescence from the nanoparticles. In this work, we did not follow the growth of any inert shell over CS (30 mol% Nd^3+^) UCNPs as our goal was to allow the excitation energy to migrate along the core/shell interface to both the core and the outermost shell to produce efficient multicolor UCL. Though Nd^3+^–Nd^3+^ energy migration is not reported in the literature, using lower doping of Nd^3+^ ions (1 mol%) in the core and outermost shell is due to two reasons: (i) to systematically follow the UCL spectra of UCNPs upon growth of shells in a layer-by-layer fashion, under single 808 nm excitation and (ii) to help the energy transfer (Nd^3+^–Yb^3+^) across the core/shell interfaces through a resonant ET process between Nd^3+^ ions.

**Fig. 7:**
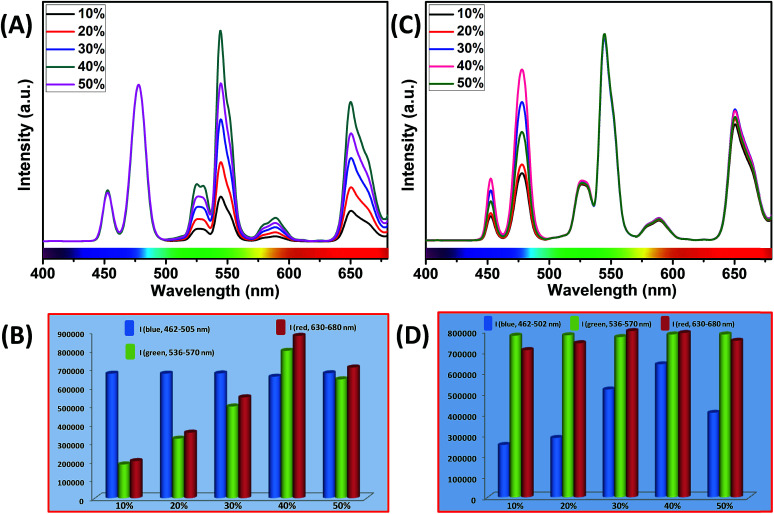
(A) UCL spectra of CSS (5 mol% Er^3+^) UCNPs with different Yb^3+^ concentrations in the outermost shell, where the Yb^3+^ concentration in the core remains unchanged at 40 mol%. Note that the UCL spectra are normalized to the blue (Tm^3+^, 462–505 nm) emission. (B) Integrated intensity of blue (Tm^3+^, 462–505 nm), green (Er^3+^, 536–570 nm) and red (Er^3+^, 630–680 nm) emissions of CSS UCNPs with different Yb^3+^ concentrations in the outermost shell. Note that integrated emissions are normalized to the blue (Tm^3+^, 462–505 nm) emission. (C) UCL spectra of CSS (5 mol% Er^3+^) UCNPs with different Yb^3+^ concentrations in the core, where the Yb^3+^ concentration in the outermost shell remains intact at 40 mol%. Note that the UCL spectra are normalized to the green (Er^3+^, 536–570 nm) emission. (D) Integrated intensity of blue (Tm^3+^, 462–505 nm), green (Er^3+^, 536–570 nm) and red (Er^3+^, 630–680 nm) emissions of CSS UCNPs with different Yb^3+^ concentrations in the core. Note that integrated emissions are normalized to the green (Er^3+^, 536–570 nm) emission.

It is interesting to note that, in the core and outermost shell, a relatively high concentration (40 mol%) of Yb^3+^ ions was used in our designed CSS (5 mol% Er^3+^) UCNPs. To examine the impact of Yb^3+^ concentration in the outermost shell on the complete UCL spectrum of these CSS UCNPs, we varied the concentration of Yb^3+^ ions in the outermost shell, while the Yb^3+^ concentration (40 mol%) in the core was kept fixed. As shown in Fig. 7A, maximum green and red emission intensities are observed with an increase in Yb^3+^ ions concentration (in the outermost shell) up to 40 mol%, whereas the intensity of blue emissions remains almost unchanged. From Fig. 7B, it is clear that the calculated integrated intensity of the green (536–570 nm) emission of CSS UCNPs with 40 mol% Yb^3+^ concentration in the outermost shell is 4.4 and 1.2 times higher than that of CSS nanoparticles with 10 and 50 mol% Yb^3+^ concentration in the outermost shell, respectively. The change in the calculated integrated intensity of red (630–680 nm) emission was similar to that of green emission.

Furthermore, we examined the impact of Yb^3+^ ions in the core on the complete UCL spectrum of our designed CSS UCNPs. We varied the concentration of Yb^3+^ ions in the core while fixing its concentration at 40 mol% in the outermost shell. As shown in Fig. 7C, as the concentration of Yb^3+^ ions is increased, the UCL intensity of blue emission increased concomitantly, reaching a maximum at 40 mol% concentration of Yb^3+^ ions, while hardly any change in the green and red emission intensity was observed.

From Fig. 7D, it is quite clear that the calculated integrated intensity of the blue (462–502 nm) emission of CSS UCNPs with 40 mol% Yb^3+^ concentration in the core is 2.5 and 1.6 times higher than that of CSS nanoparticles with 10 and 50 mol% Yb^3+^ concentrations in the core, respectively. In fact, the use of a high concentration of Yb^3+^ ions is expected to strongly affect the UCL intensity of the nanoparticles as Yb^3+^-mediated sub-lattice enables excitation energy migration from the primary sensitizer to the activator. Upon increase in Yb^3+^concentration, the total absorption cross-section of the same increases which again leads to the enhanced efficiency of ET from Nd^3+^ (middle layer domain) to Tm^3+^ (core region) and Er^3+^ (outermost shell). Moreover, a higher concentration of Yb^3+^ ions resulted in shorter Yb^3+^–Yb^3+^ interionic distance within the sublattice ensuring an efficient ET from Nd^3+^ to both the Tm^3+^-doped core and Er^3+^-doped outermost shell in CSS UCNPs. To our knowledge, literature reports suggest that the ET rate is proportional to *r*^−6^ (where, *r* is the average donor–acceptor distance) within the dipole interaction.^58,59^ Based on this fundamental, we assume that a suitable shell thickness (2.5 nm for CS and 3.5 nm for CSS, in our work) and high concentration of Nd^3+^ ions in the middle layer along with a high concentration of Yb^3+^ ions in both the core and outermost shell not only allow our designed UCNPs to migrate excitation energy directionally across the core/shell interface but also prevent the excitation energy from travelling long Nd^3+^–Nd^3+^, Yb^3+^–Yb^3+^ and Nd^3+^–Yb^3+^ distances. However, when the Yb^3+^–Yb^3+^ interionic distance exceeds the optimal value, back ET might occur from Tm^3+^ or Er^3+^ to Yb^3+^ ions, leading to the reduction of the UCL intensity from the nanoparticles. In this point of view, a high concentration of Yb^3+^ ions played a pivotal role in limiting the possibility of producing unwanted cross-relaxation pathways, while also affecting the process of bridging efficient ET from Nd^3+^ (middle layer) to Tm^3+^ (core region) and Er^3+^ (outermost shell) to produce efficient multicolor UCL from CSS UCNPs. Now, to check whether the use of a higher concentration of Yb^3+^ ions leads to the formation of bigger sized nanoparticles and simultaneously affect the UCL intensity of the same, we performed TEM measurements of core nanoparticles where the Yb^3+^ concentration was varied from 20 to 50 mol% while the concentration of other ions was kept unchanged. Almost no change in the size of the core nanoparticles was observed (Fig. S10†). Hence, from Fig. 7, we believe that the change in the UCL intensity of CSS (5 mol% Er^3+^) UCNPs is not related to the variation in size of the core but with the variation of Yb^3+^ ions in the core as well as in the outermost shell.

In addition, in our work, the Ca^2+^ ion has been chosen as dopant in each domain as the UCL intensity (438–503, blue emission) of the CS (30 mol% Nd^3+^) UCNPs with 7 mol% Ca^2+^ (in both core and shell) showed an around 1.4 times enhancement as compared to the same sample prepared without Ca^2+^ (Fig. S11†).

Very small sized NaGdF_4_ UCNPs possess a very high surface-to-volume ratio and, at the same time, a large portion of dopant ions occupy positions at the surface of the nanoparticles. Thus, the excitation energy is easily quenched either by surface defects or surface capping ligands. Therefore, it is essential to modify the host lattice and this is possible while Ca^2+^ ions are introduced into the NaGdF_4_ core (and even into the shell). Due to the lower valence, when Ca^2+^ is introduced to replace Gd^3+^, a charge imbalance occurs in NaGdF_4_. In addition, F^−^ vacancies play an important role in charge compensation inside the crystal lattice. This induces the formation of transient electric dipoles with positive poles pointing outward on the grain surfaces. These transient electric dipoles accelerate the diffusion of ions for the crystal growth, needing F^−^ ions from the solution to the grain and thus promoting the growth of NaGdF_4_. In this case, the growth of NaGdF_4_ nanoparticles is indeed a diffusion-controlled process, resulting in better size-uniformity and fewer surface defects which lead to the enhancement of the UCL intensity of the UCNPs. Moreover, the formation of F^−^ vacancies induced by the substitution of Gd^3+^ with Ca^2+^ in the host lattice lowers the local crystal field symmetry around Yb^3+^, Nd^3+^, Er^3+^ and Tm^3+^ ions, which in turn affects the intra 4f–4f transition possibility of Ln^3+^ ions and subsequently enhances the UCL intensity from the CSS UCNPs.

**Fig. 8:**
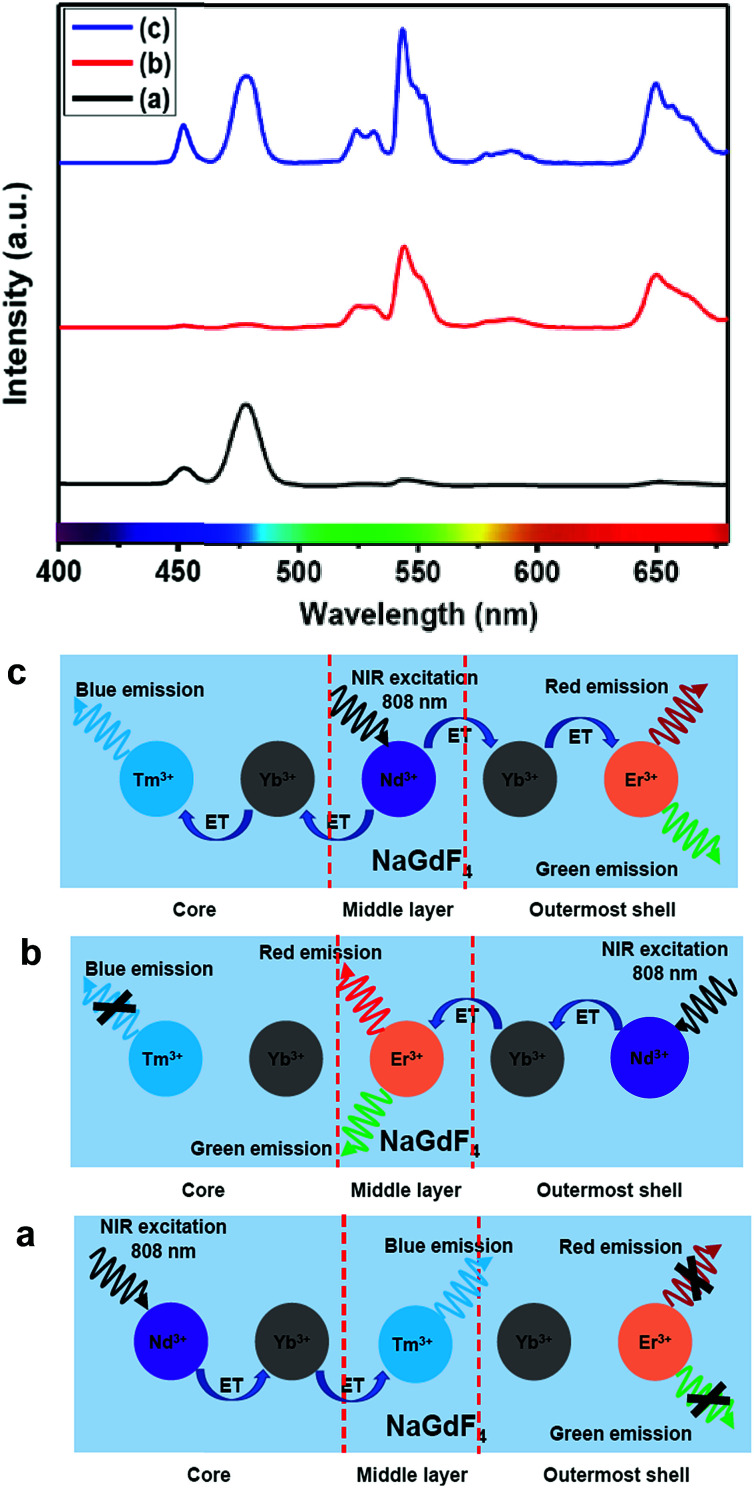
UCL spectra of (a) NaGdF_4_:Nd^3+^(30)/Ca^2+^(7)@NaGdF_4_:Nd^3+^(1)/Ca^2+^(7)/Tm^3+^(0.75)/Yb^3+^(40)@NaGdF_4_:Yb^3+^(40)/Nd^3+^(1)/Ca^2+^(7)/Er^3+^(5), (b) NaGdF_4_:Tm^3+^(0.75)/Yb^3+^(40)/Nd^3+^(1)/Ca^2+^(7)@NaGdF_4_:Nd^3+^(1)/Ca^2+^(7)/Yb^3+^(40)/Er^3+^(5)@NaGdF_4_:Nd^3+^(30)/Ca^2+^(7) and (c) NaGdF_4_:Tm^3+^(0.75)/Yb^3+^(40)/Nd^3+^(1)/Ca^2+^(7)@NaGdF_4_:Nd^3+^(30)/Ca^2+^(7)@NaGdF_4_:Yb^3+^(40)/Nd^3+^(1)/Ca^2+^(7)/Er^3+^(5) core/shell/shell UCNPs (808 nm laser excitation). Schematic illustration of the switching of the Nd^3+−^ enriched domain to emit multicolor UCL from corresponding core/shell/shell UCNPs.

Furthermore, to shed light on the position of the Nd^3+^-enriched harvesting layer (*i.e.*, first shell or middle layer) in the CSS (5 mol% Er^3+^) UCNPs, we synthesized NaGdF_4_:Nd^3+^(30)/Ca^2+^(7)@NaGdF_4_:Nd^3+^(1)/Ca^2+^(7)/Tm^3+^(0.75)/Yb^3+^(40)@NaGdF_4_:Yb^3+^(40)/Nd^3+^(1)/Ca^2+^(7)/Er^3+^(5) CSS and NaGdF_4_:Tm^3+^(0.75)/Yb^3+^(40)/Nd^3+^(1)/Ca^2+^(7)@NaGdF_4_:Nd^3+^(1)/Ca^2+^(7)/Yb^3+^(40)/Er^3+^(5)@NaGdF_4_:Nd^3+^(30)/Ca^2+^(7) CSS UCNPs, whereas the Nd^3+^-enriched domain was placed in the core and outermost shell, respectively, and the energy transfer process (Nd^3+^ → Yb^3+^ → Er^3+^/Tm^3+^) was uni-directional. Fig. 8 clearly depicts that the UCL [blue or (red + green)] is only generated from Tm^3+^ or Er^3+^ ions from those CSS UCNPs. On the other hand, the Nd^3+^-enriched middle shell along with separation of Tm^3+^ (in core) and Er^3+^ ions (in outermost shell) enable the CSS UCNPs to produce efficient multicolor UCL (Fig. 8c). These results suggest that the ET in a bi-directional manner dominates the uni-directional one to achieve efficient multicolor UCL without implication of any deleterious cross-relaxation mechanism between the activator ions.

**Fig. 9:**
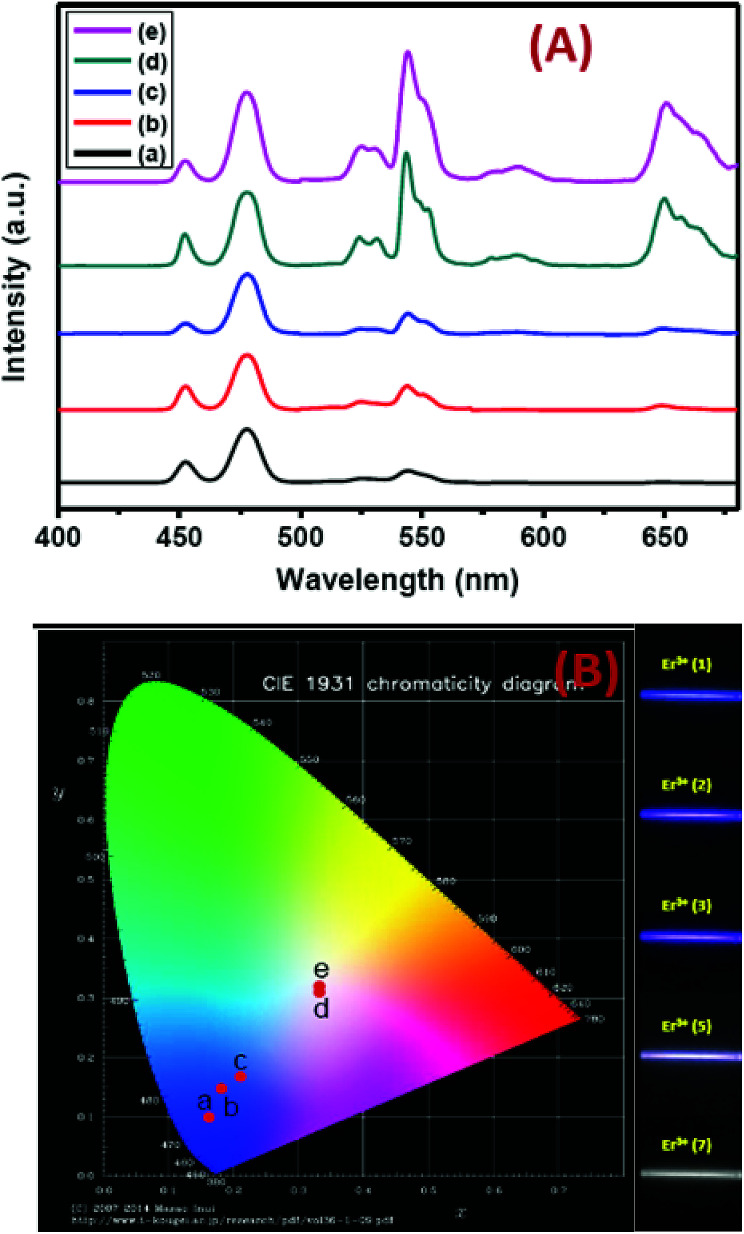
(A) UCL spectra (B) corresponding ‘Commission Internationale de l’Eclairage’ (CIE) and digital images of CSS UCNPs. a, b, c, d and e in the CIE diagram indicate the corresponding UCNPs, having Er^3+^ concentrations 1, 2, 3, 5 and 7 mol%, respectively, in the outermost shell. Digital images are taken using an 808 nm laser, 10 W cm^−2^ (without using any filter).

Precise control of the concentration of Er^3+^ (activator) ions in the outermost shell of CSS UCNPs makes them potential candidates to produce efficient multicolor UCL output. There is hardly any change in the size (Fig. S12†) and size distribution (Fig. S13†) observed for CSS UCNPs with varying Er^3+^ concentration in the outermost shell. Moreover, it is evident from Fig. 9A that the appropriate tuning of the concentration of Er^3+^ ions in the outermost shell plays a vital role in the change in relative intensities of the green (^4^S_3/2_ → ^4^I_15/2_) and red (^4^F_9/2_ → ^4^I_15/2_) emissions of Er^3+^ ions, resulting in the continuous production of UCL colors ranging from blue to white. Such spectral changes could also be observed by the naked eye (Fig. 9B, right side). Furthermore, the calculated chromaticity coordinates (Fig. 9B) reveal a similar trend of the emission colors as perceived through spectroscopic study and naked eye visualization. It is worth mentioning that control in the use of each activator (*e.g.*, Tm^3+^ or Er^3+^) in the CSS UCNPs allows manipulating and producing different UCL spectra with different colors. From Fig. S14,† it is quite clear that the core/shell/shell UCNPs (analogues to CSS and without Er^3+^ in the outermost shell) show pure blue emission at 451 and 477 nm from Tm^3+^ ions positioned in the core. However, core/shell/shell UCNPs (analogues to CSS and without Tm^3+^ in the core) exhibit both green (540 nm) and red (650 nm) emissions, which originate from Er^3+^ ions present in the outermost shell. On the other hand, our designed CSS (5 mol% Er^3+^) shows a three primary color (blue, green and red) blended spectrum responsible for generating white UCL light.

To understand whether the middle shell layer-to-core (Nd^3+^ to Tm^3+^) ET or the middle shell layer-to-outermost shell (Nd^3+^ to Er^3+^) ET has higher contribution to the overall UCL to produce multicolor emission, as a control experiment, Tm^3+^ ions in the core and Er^3+^ ions in the shell of the CSS (5 mol% Er^3+^) were subjected to the corresponding direct excitations at 357 and 488 nm, respectively. As shown in Fig. S15,† the integrated PL emission intensity of Tm^3+^ ions (*λ*_exi_ = 357 nm) was slightly higher (1.3 times) than that of Er^3+^ ions (*λ*_exi_ = 488 nm). As the absorption coefficients of Tm^3+^ and Er^3+^ ions are lower and similar (∼2–10 M^−1^ cm^−1^),^60^ we may conclude that Tm^3+^ excited states favour a radiative decay pathway slightly more compared to that of Er^3+^ ions. However, the ratio between the integrated area of green + red (510–570 nm + 630–680 nm, Er^3+^) and blue (436–500 nm, Tm^3+^) regions in the UCL spectrum of the same CSS UCNPs was found to be 2.4 indicating higher contribution from Er^3+^. This is only possible if the ET from Nd^3+^ to Er^3+^ is more compared to Nd^3+^ to Tm^3+^ in the UCNPs.

Furthermore, to investigate the number of photons involved to produce white UCL, the power dependence of UCL was measured from CSS (5 mol% Er^3+^) UCNPs. Fig. S16† shows that the number of photons involved in multicolor UCL is 3 (slope, 3.01), 2 (slope, 1.98) and 2 (slope, 1.76) for blue, green and red emissions, respectively. These results are in good agreement with the literature reports.

## Conclusion

In summary, we have successfully illustrated multicolor UCL from Nd^3+^-sensitized Gd^3+^-based core/shell/shell UCNPs under biologically relevant, single wavelength excitation (808 nm) which, unlike 980 nm excitation, does not cause biological heating problems that could damage cells and tissues. The separation of Tm^3+^ (in the core) and Er^3+^ (in the outermost shell layer) in the core/shell/shell UCNPs effectively alleviates the deleterious cross-relaxation processes between them. The doping of a Nd^3+^ sensitizer (30 mol%) in the middle layer, on the other hand, enables efficient harvesting of excitation light and concurrent migration of the energy across the core/shell interfaces to both Tm^3+^ in the core and Er^3+^ in the outermost shell layer. Precise control of the shell thickness and judicious manipulation of Ln^3+^ ions (activators and sensitizers) along with their spatial distribution in their defined layer domain provide a broad range of multicolor UCL (from blue to white) through a bi-directional ET process. Such tuneable multicolor UCL makes these sub-20 nm sized Nd^3+^-sensitized Gd^3+^-based core/shell/shell UCNPs potential candidates for bioimaging and MRI dual modal applications.

## Conflicts of interest

There are no conflicts to declare.

## Supplementary Material

NA-001-C9NA00006B-s001
