# Supplementary material for: Precisely tailored shell thickness and Ln3+ content to produce multicolor emission from Nd3+-sensitized Gd3+-based core/shell/shell UCNPs through bi-directional energy transfer
Source: Nanoscale Adv. 2019 Mar 21;1(5):1936–47. doi: 10.1039/c9na00006b (PMC9418845; doi:10.1039/c9na00006b)
Supplement: NA-001-C9NA00006B-s001 [file NA-001-C9NA00006B-s001.pdf]

Electronic Supplementary Material (ESI) for Nanoscale Advances.
This journal is © The Royal Society of Chemistry 2019

Electronic Supporting Information

Precisely Tailored Shell Thickness and Ln^{3+} Content to Produce Multicolor Emission from Nd^{3+}-Sensitized Gd^{3+}-Based Core/Shell/Shell UCNPs Through Bi-directional Energy Transfer

York E. Serge Correales,‡[a] Chanchal Hazra,‡[a*] Sajjad Ullah,[b] Laís R. Lima[a] and Sidney J. L. Ribeiro [a*]

[a]Institute of Chemistry, São Paulo State University, UNESP, 14800-060, Araraquara, SP, Brazil

[b]Institute of Chemical Sciences, University of Peshawar, 25120, Peshawar, Pakistan

Corresponding Author: sidney.jl.ribeiro@unesp.br or sjlribeiro@gmail.com and chanchalhazra007@gmail.com

Table of contents

Calculation of UCL intensity (integrated are) of the blue, green and red emissions against different thickness of the outermost shell of core/shell/shell UCNPs..............2

Histogram of core, core/shell and core/shell/shell UCNPs.. 2

High resolution of TEM images of core, core/shell and core/shell/shell UCNPs.......... 3

EDS spectra of core, core/shell and core/shell/shell UCNPs... 4

Comparison of IR spectra of free oleic acid and oleic acid capped UCNPs.......................5

Absorption spectra of core, core/shell and core/shell/shell UCNPs................................ 6

UCL spectra of core/shell/shell UCNPs (without normalization) with different thickness of outermost shell...7

Optimization in the concentration of Tm^{3+} ions in core/shell UCNPs...8

Optimization of Nd^{3+} ion concentration in the shell of core/shell UCNPs.........................9

Crystalline phase and size investigation of core/shell UCNPs with different concentration of Nd^{3+} ions in the shell..10

TEM images of core UCNPs with different concentration of Yb^{3+} ions................................11

UC comparison of Ca^{2+}-doped and un-doped core/shell UCNPs..12

TEM images of core/shell/shell UCNPs with different Er^{3+} concentration in the outermost shell... 13

Histogram of core/shell/shell UCNPs with different Er^{3+} concentration in the outermost shell.. 14

UCL Comparison of Tm^{3+}-doped, Er^{3+}-doped and Tm^{3+}/Er^{3+} co-doped core/shell/shell UCNPs....................15

PL emission spectra of core/shell/shell nanoparticles using direct excitation of Tm^{3+} ion and Er^{3+} ion....................16

Calculation of photons involved in the white UCL....................17

Table S1. Calculation of UCL intensity (integrated area) of the blue, green and red emissions against different thickness of the outermost shell of CSS (5 mol % Er^{3+}) [$NaGdF_4$:Tm^{3+}(0.75)/Yb^{3+}(40)/Nd^{3+}(1)/Ca^{2+}(7)@$NaGdF_4$:Nd^{3+}(30)/Ca^{2+}(7)@$NaGdF_4$:Yb^{3+}(40)/Nd^{3+}(1)/Ca^{2+}(7)/Er^{3+}(5)] UCNPs.

**Outermost shell thickness**	**Intensity (462-505 nm)**	**Intensity (536-570 nm)**	**Intensity (630-680 nm)**
1.2 nm	649492.32	350358.44	385910.01
2.5 nm	646365.80	612980.09	676026.64
3.5 nm	668118.19	811079.78	894514.75
5 nm	648936.87	476545.11	524901.11
5.7 nm	658374.04	178068.93	9228.86

Histogram of core, core/shell and core/shell/shell UCNPs

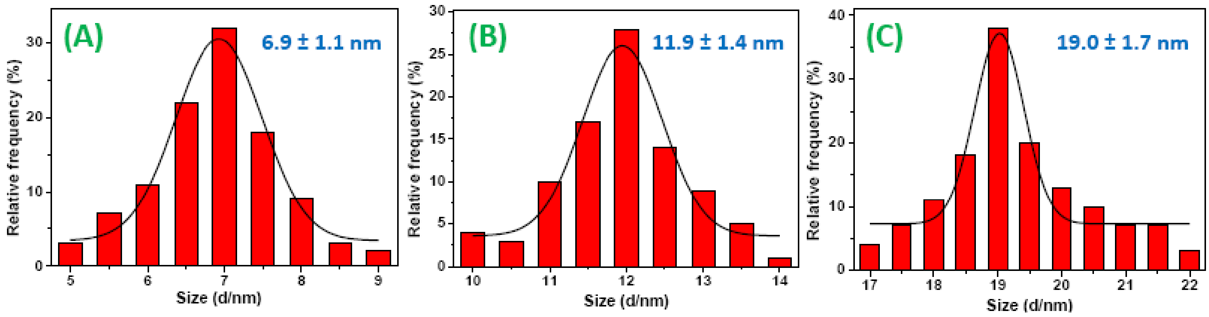

Fig. S1. Size distribution of (A) core $NaGdF_4$:Tm^{3+}(0.75)/Yb^{3+}(40)/Nd^{3+}(1)/Ca^{2+}(7) (B) CS (30 mol % Nd^{3+}) [$NaGdF_4$:Tm^{3+}(0.75)/Yb^{3+}(40)/Nd^{3+}(1)/Ca^{2+}(7)@$NaGdF_4$:Nd^{3+}(30)/Ca^{2+}(7)] and (C) CSS (5 mol % Er^{3+}) [$NaGdF_4$:Tm^{3+}(0.75)/Yb^{3+}(40)/Nd^{3+}(1)/Ca^{2+}(7)@$NaGdF_4$:Nd^{3+}(30)/Ca^{2+}(7)@$NaGdF_4$:Yb^{3+}(40)/Nd^{3+}(1)/Ca^{2+}(7)/Er^{3+}(5)] UCNPs. Statistical analysis was performed on 100 particles in each of the two batches, recurring the respective size distributions of the corresponding nanoparticles. Average diameter (d/nm) of the corresponding core, core/shell and core/shell/shell UCNPs is shown with significant error.

High resolution TEM images of core, core/shell and core/shell/shell UCNPs

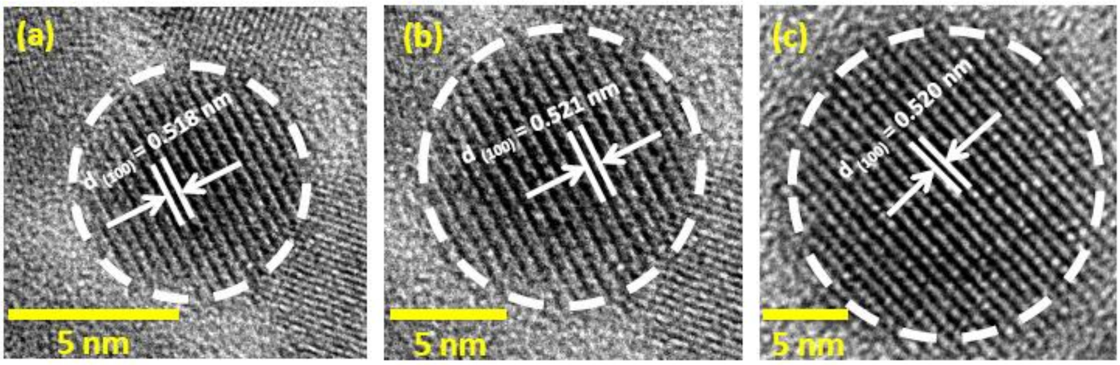

Fig. S2. High resolution TEM (HRTEM) images of (a) core $NaGdF_4$:Tm^{3+}(0.75)/Yb^{3+}(40)/Nd^{3+}(1)/Ca^{2+}(7) (b) CS (30 mol % Nd^{3+}) [$NaGdF_4$:Tm^{3+}(0.75)/Yb^{3+}(40)/Nd^{3+}(1)/Ca^{2+}(7)@$NaGdF_4$:Nd^{3+}(30)/Ca^{2+}(7)] and (c) CSS (5 mol % Er^{3+}) [$NaGdF_4$:Tm^{3+}(0.75)/Yb^{3+}(40)/Nd^{3+}(1)/Ca^{2+}(7)@$NaGdF_4$:Nd^{3+}(30)/Ca^{2+}(7)@$NaGdF_4$:Yb^{3+}(40)/Nd^{3+}(1)/Ca^{2+}(7)/Er^{3+}(5)] UCNPs. The HRTEM images present the lattice fringes with an observed d-spacing of ~0.52 nm, which is in agreement with the lattice spacing in the (100) plane of hexagonal-phase $NaGdF_4$, indicating a high crystallinity of the corresponding core, core/shell and core/shell/shell nanoparticles.

EDS spectra of core, core/shell and core/shell/shell UCNPs

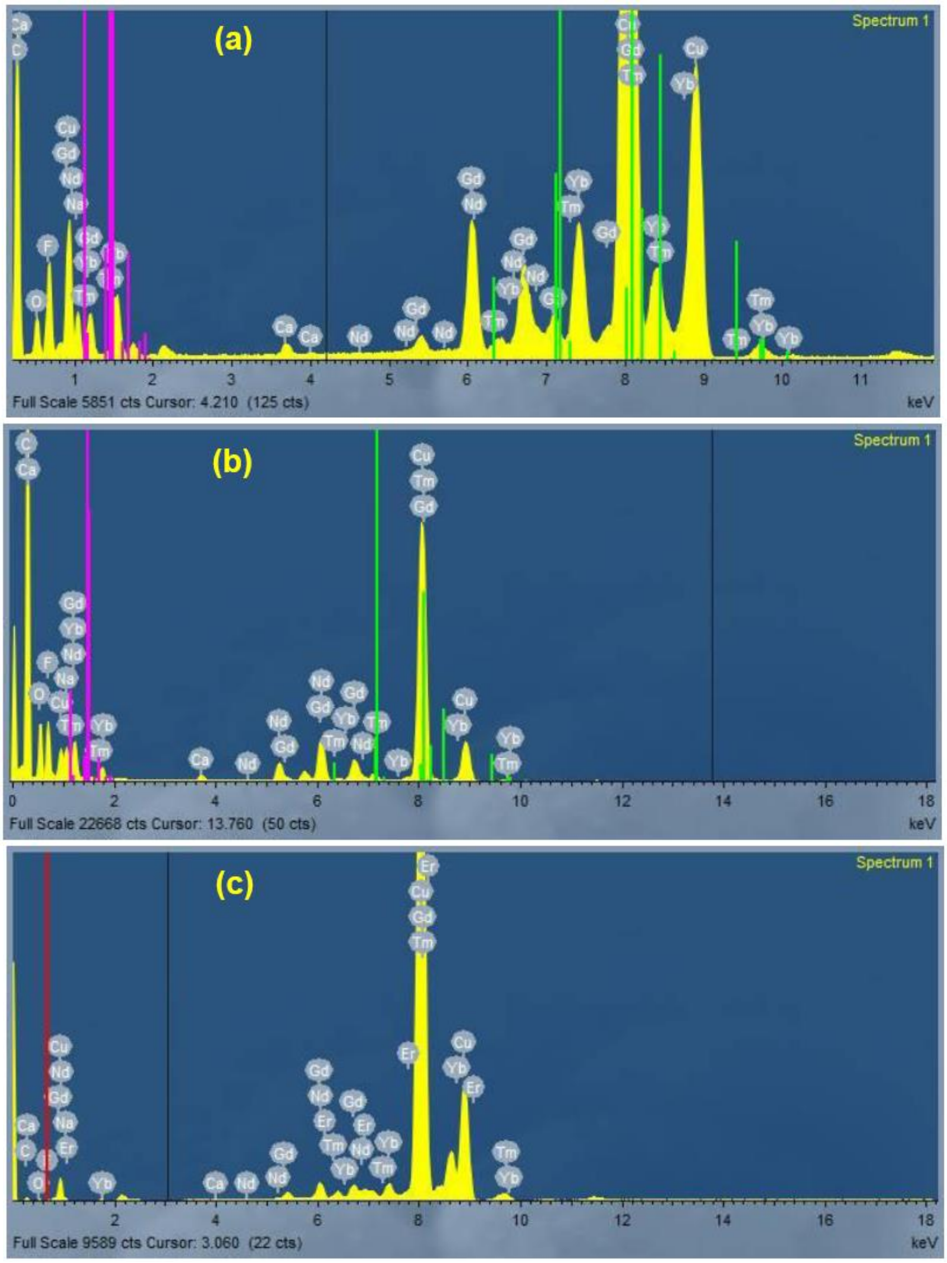

Fig. S3. EDS spectra of (a) core $NaGdF_4$:Tm^{3+}(0.75)/Yb^{3+}(40)/Nd^{3+}(1)/Ca^{2+}(7) (b) CS (30 mol % Nd^{3+}) [$NaGdF_4$:Tm^{3+}(0.75)/Yb^{3+}(40)/Nd^{3+}(1)/Ca^{2+}(7)@$NaGdF_4$:Nd^{3+}(30)/Ca^{2+}(7)] and (c) CSS (5 mol % Er^{3+}) [$NaGdF_4$:Tm^{3+}(0.75)/Yb^{3+}(40)/Nd^{3+}(1)/Ca^{2+}(7)@$NaGdF_4$:Nd^{3+}(30)/Ca^{2+}(7)@$NaGdF_4$:Yb^{3+}(40)/Nd^{3+}(1)/Ca^{2+}(7)/Er^{3+}(5)] UCNPs. The spectra show the presence of (Gd, Nd, Ca, Yb, Tm), (Gd, Nd, Ca, Yb, Tm) and (Gd, Nd, Ca, Yb, Tm, Er) for core, core/shell and core/shell/shell UCNPs, respectively, suggesting the growth of shells on core nanoparticles layer-by-layer. In each spectrum, Cu element appears due to the use of carbon coated cupper grid during TEM measurements.

Comparison of IR spectra of free oleic acid and oleic acid capped UCNPs

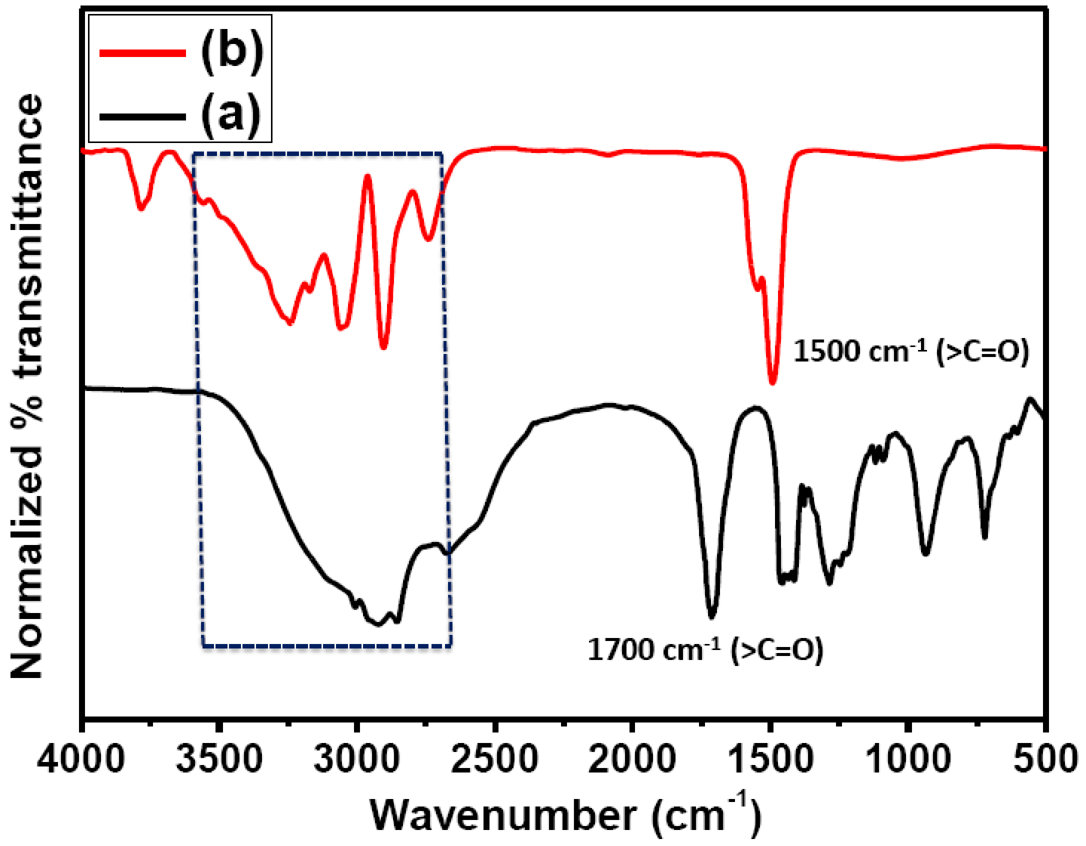

Fig. S4. FTIR spectra of (a) pure oleic acid molecules and (b) oleic acid capped CSS (5 mol % Er^{3+}) [$NaGdF_4$:Tm^{3+}(0.75)/Yb^{3+}(40)/Nd^{3+}(1)/Ca^{2+}(7)@$NaGdF_4$:Nd^{3+}(30)/Ca^{2+}(7)@$NaGdF_4$:Yb^{3+}(40)/Nd^{3+}(1)/Ca^{2+}(7)/Er^{3+}(5)] UCNPs. Selected area indicates the presence of stretching vibration of $-CH_2$ units in oleic acid. Decrease in the broadness of $-CH_2$ stretching vibration also indicates the strong attachment of oleic acid molecules onto the surface of the core/shell/shell UCNPs.

Absorption spectra of core, core/shell and core/shell/shell UCNPs

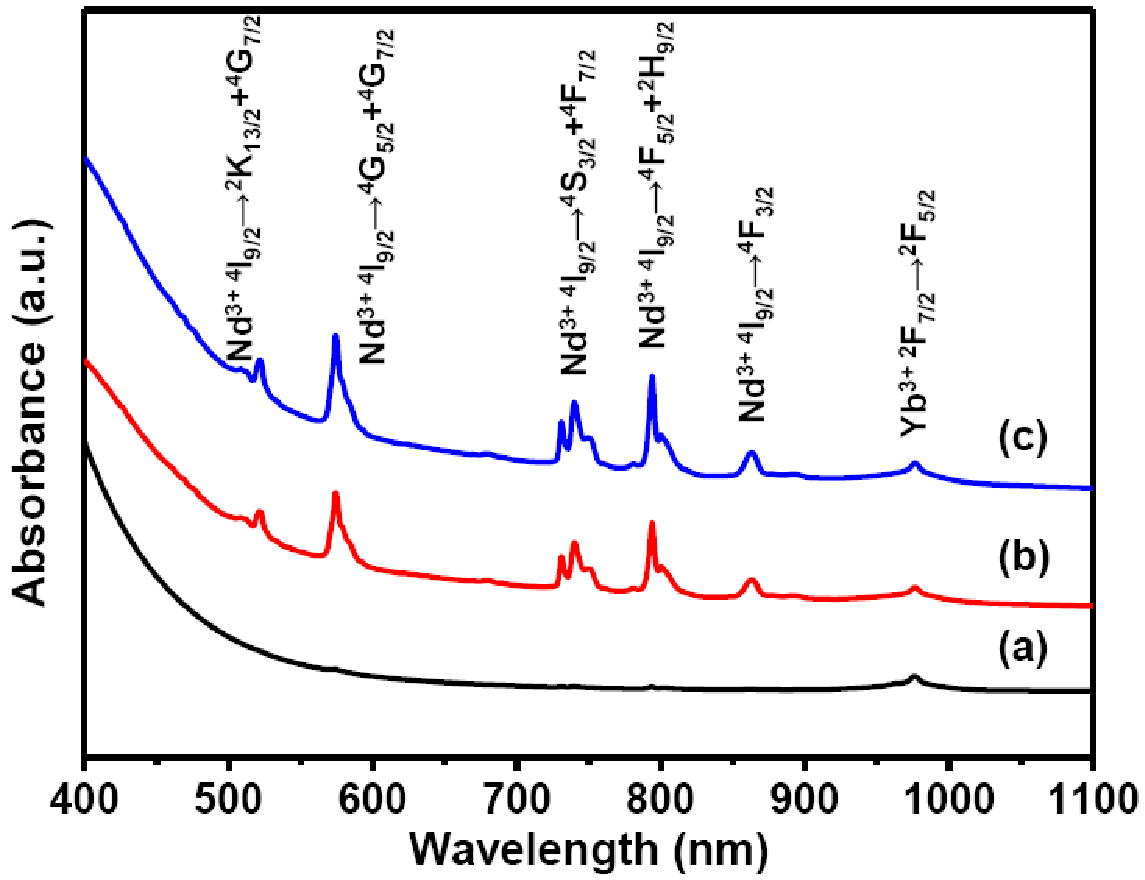

Fig. S5. The absorption spectra of the (a) core $NaGdF_4$:Tm^{3+}(0.75)/Yb^{3+}(40)/Nd^{3+}(1)/Ca^{2+}(7) (b) CS (30 mol % Nd^{3+}) [$NaGdF_4$:Tm^{3+}(0.75)/Yb^{3+}(40)/Nd^{3+}(1)/Ca^{2+}(7)@$NaGdF_4$:Nd^{3+}(30)/Ca^{2+}(7)] and (c) CSS (5 mol % Er^{3+}) [$NaGdF_4$:Tm^{3+}(0.75)/Yb^{3+}(40)/Nd^{3+}(1)/Ca^{2+}(7)@$NaGdF_4$:Nd^{3+}(30)/Ca^{2+}(7)@$NaGdF_4$:Yb^{3+}(40)/Nd^{3+}(1)/Ca^{2+}(7)/Er^{3+}(5)] UCNPs. Absorption peak around 980 nm was observed, assigning to the ground state absorption of the $^2F_{7/2}\rightarrow{}^2F_{5/2}$ transition of Yb^{3+} ions. A strong absorption peak (for b and c) at ~800 nm was observed, corresponding to the ground state absorption of the $^4I_{9/2}\rightarrow{}^4F_{5/2}/{}^2H_{9/2}$ transition of Nd^{3+} ions. Very small absorption peak (for a) at ~800 nm (i.e. $^4I_{9/2}\rightarrow{}^4F_{5/2}/{}^2H_{9/2}$ transition of Nd^{3+} ions) was observed, indicating the presence of small 1 mol% of Nd^{3+} ions in the core nanoparticles.

UCL spectra of core/shell/shell UCNPs (without normalization) with different thickness of outermost shell

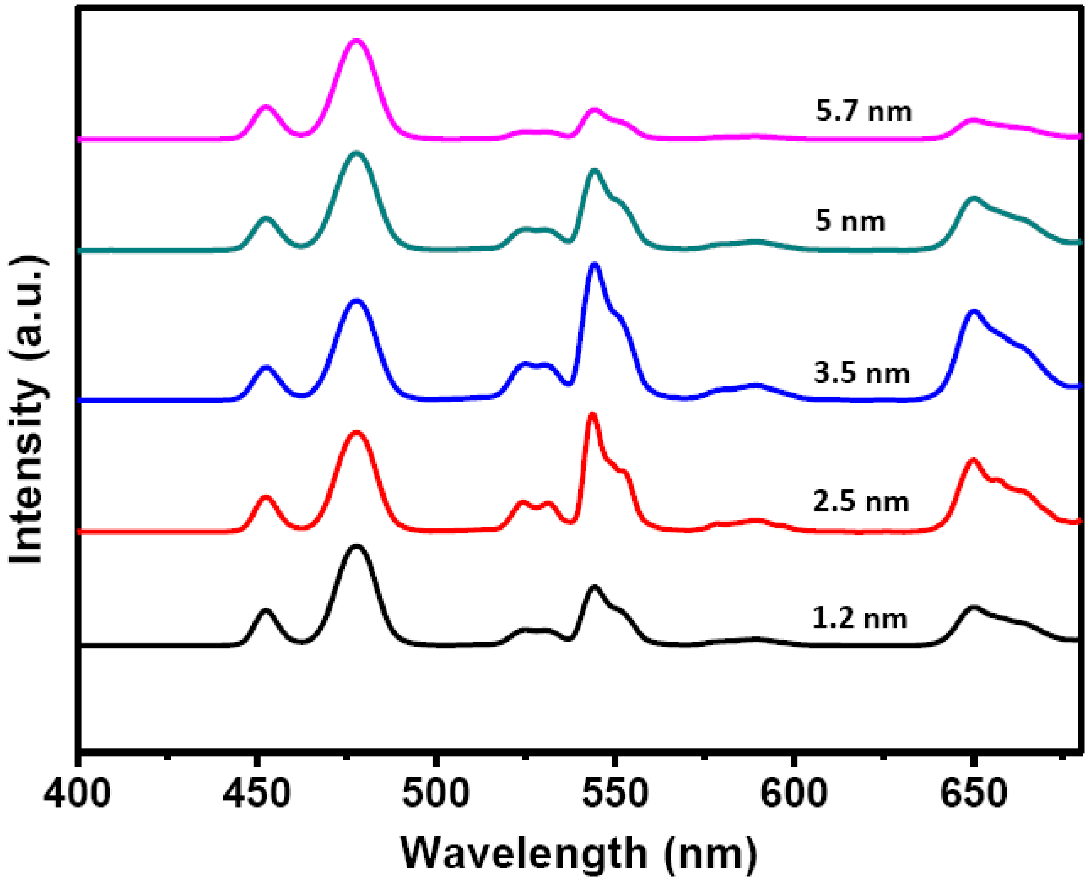

Fig. S6. UCL spectra (without normalization) of CSS UCNPs (5 mol % Er^{3+}) with different thickness of outermost shell over CS (30 mol % Nd^{3+}) UCNPs (d = 1.2 – 5.7 nm).

Optimization in the concentration of Tm^{3+} ions in core/shell UCNPs

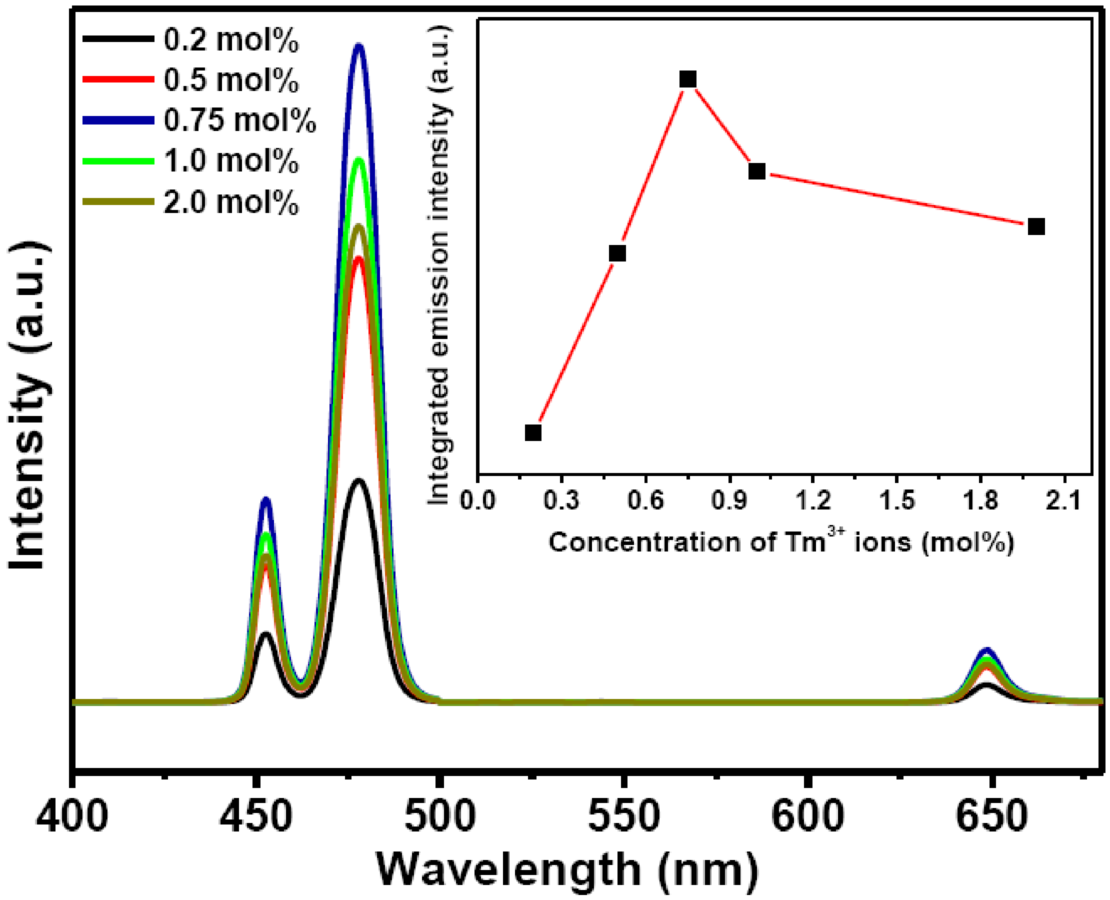

Fig. S7. Optimization in the concentration of Tm^{3+} ions (against 40 mol% Yb^{3+} ion) in the CS (30 mol % Nd^{3+}) [$NaGdF_4$:Tm^{3+}(0.75)/Yb^{3+}(40)/Nd^{3+}(1)/Ca^{2+}(7)@$NaGdF_4$:Nd^{3+}(30)/Ca^{2+}(7)] UCNPs. The optimized concentration of Tm^{3+} ions leads to produce strongest UCL from the core nanoparticles. Beyond the optimized concentration of Tm^{3+} ions, the integrated emission intensity decreases because of the self-quenching of the activator (i.e. Tm^{3+}) ions.

Optimization of Nd^{3+} ion concentration in the shell of core/shell UCNPs

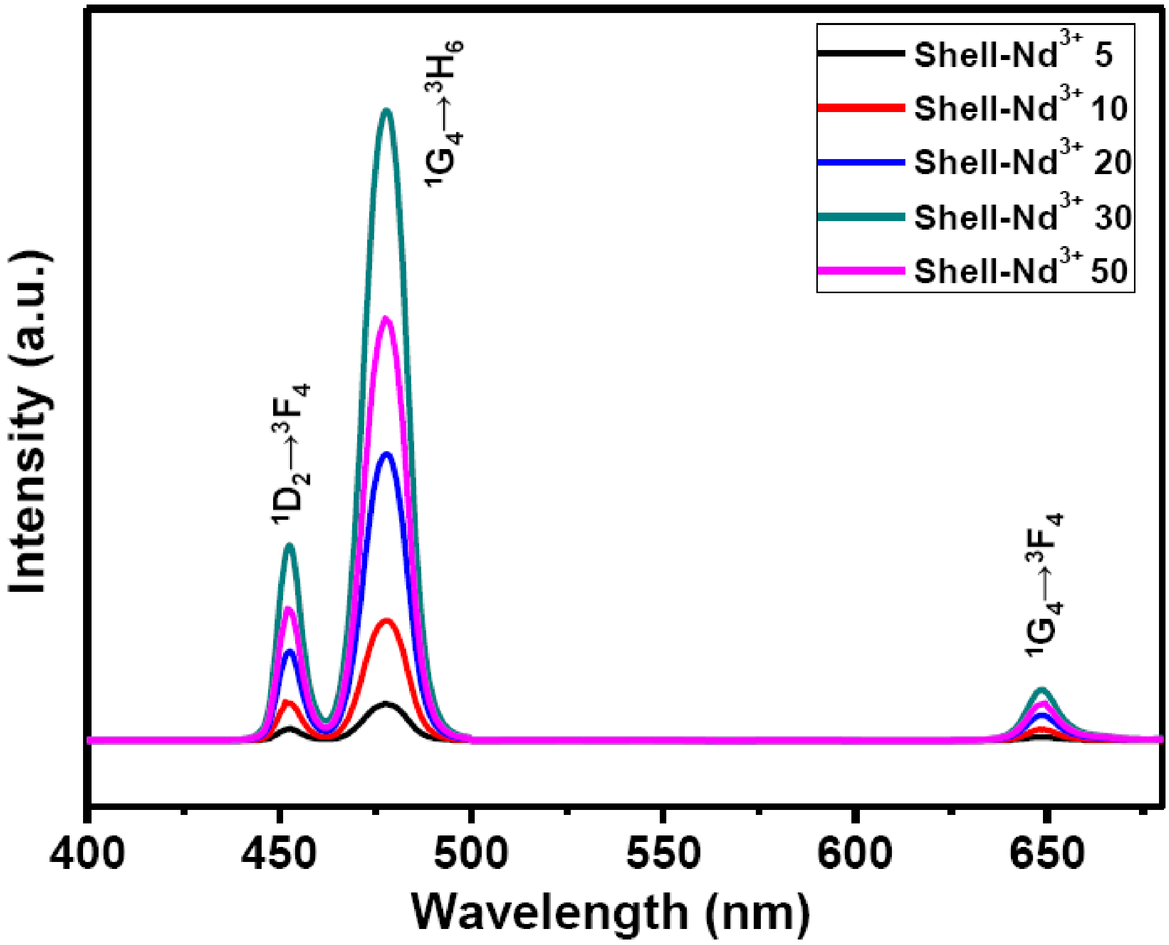

Fig. S8. The UCL spectra from CS (Nd^{3+} = 5, 10, 20, 30 and 50 mol %) UCNPs. The optimized Nd^{3+} content was determined to be 30 mol%. Upon 808 nm excitation, the integrated UCL intensity at ~477 nm of the $^1G_4 \rightarrow ^3H_6$ of Tm ions from CS (30 mol % Nd^{3+}) UCNPs enhanced by 17.5 times, compared with the sample doped with 5 mol % Nd^{3+} in the shell.

Crystalline phase and size investigation of core/shell UCNPs with different concentration of Nd^{3+} ions in the shell

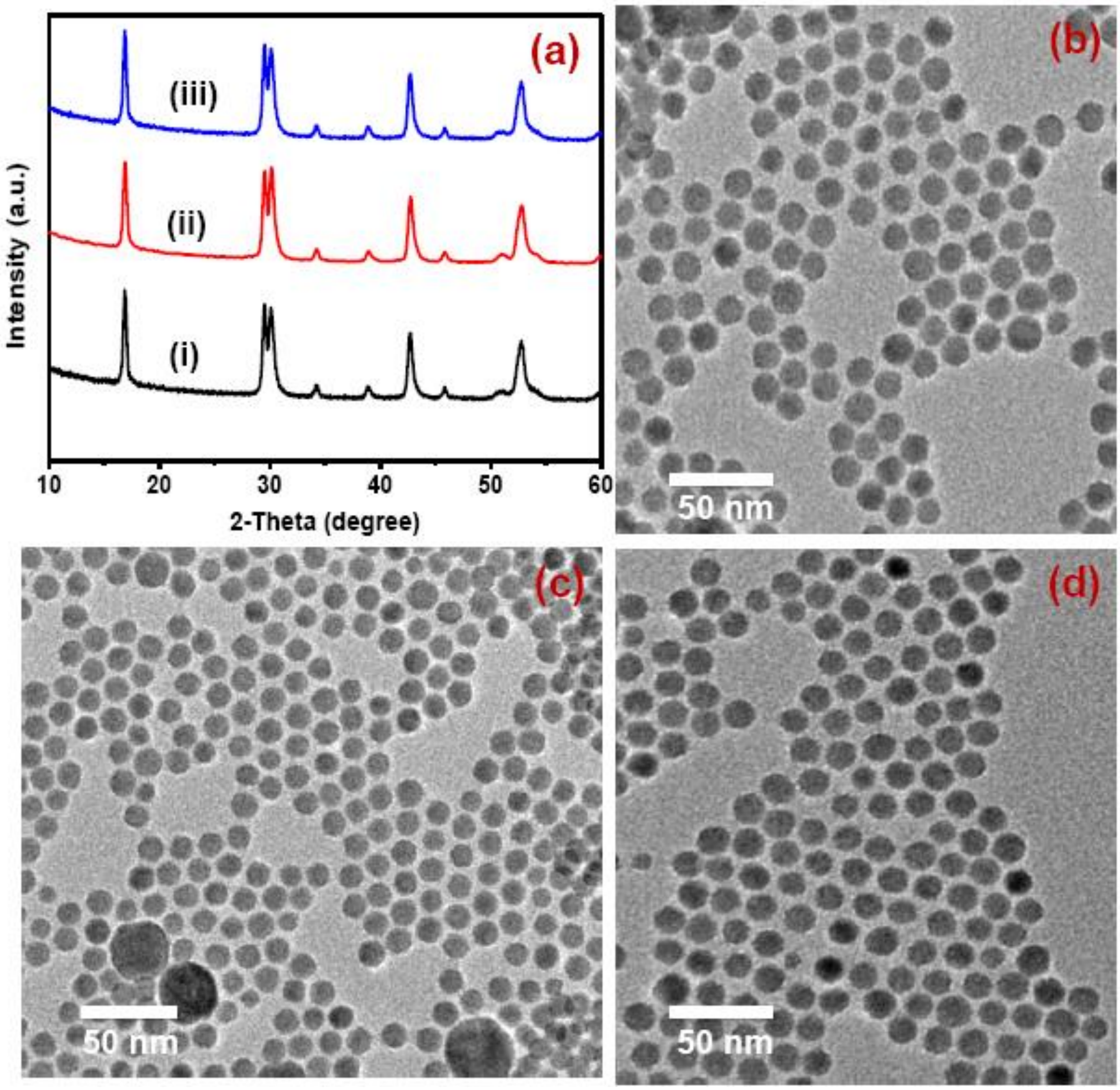

Fig. S9. (a) XRD of CS (Nd^{3+} = 10, 20 and 50 mol %) UCNPs [10 (i), 20 (ii) and 50 mol % (iii)]. The core/shell nanoparticles with different Nd^{3+} concentration in the shell remain intact along with hexagonal phase (JCPDS No: 27-0699). TEM images of CS (Nd^{3+} = 10, 20 and 50 mol %) UCNPs [10 (b), 20 (c) and 50 mol % (d)]. From these images, it is clearly noted that the all as-prepared core/shell UCNPs have uniform spherical shape with an average diameter ~12 nm.

TEM images of core UCNPs with different concentration of Yb^{3+} ions

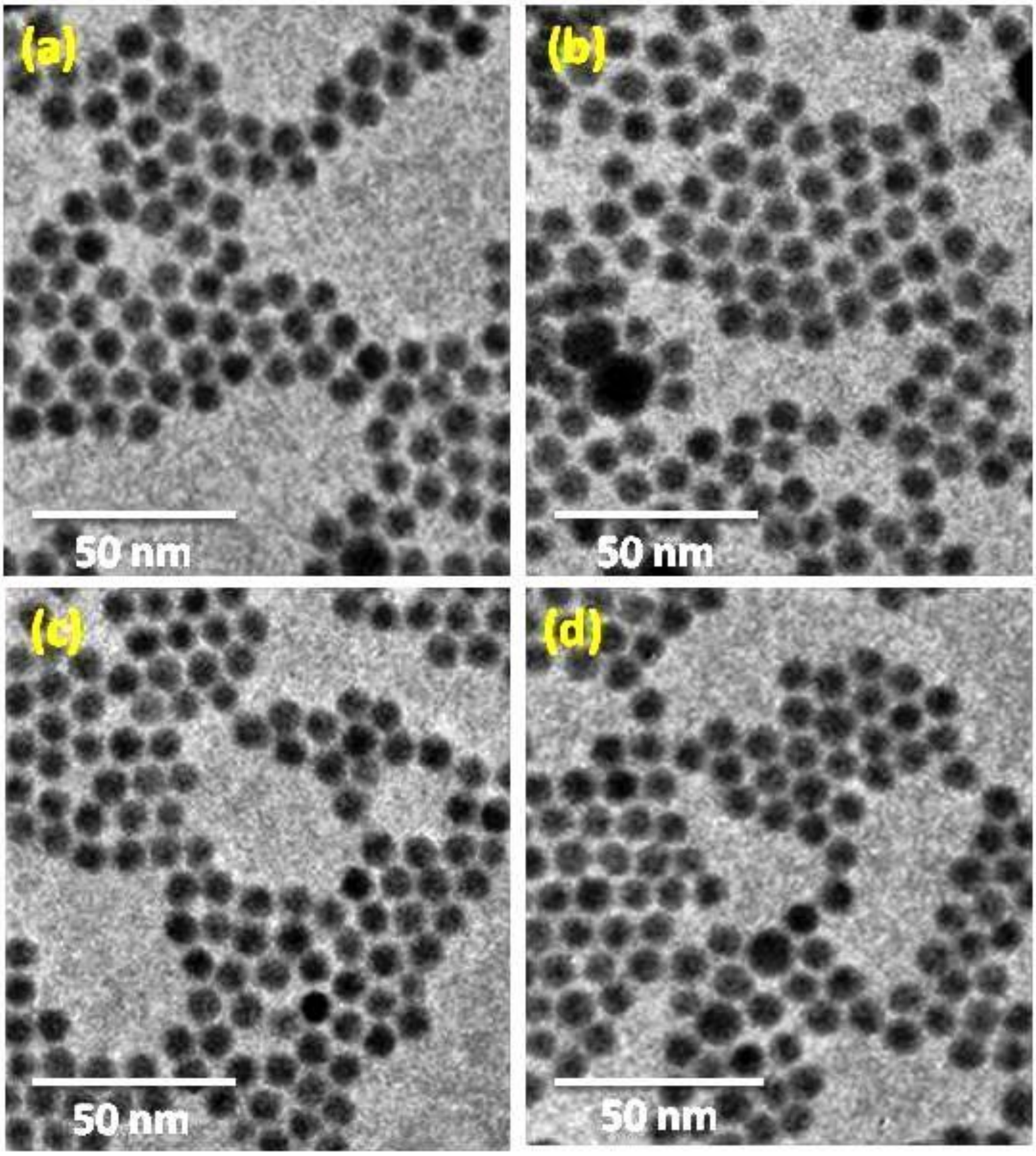

Fig. S10. TEM images of core $NaGdF_4$:Tm^{3+}(0.75)/Yb^{3+}(40)/Nd^{3+}(1)/Ca^{2+}(7) UCNPs with different (a – 10, b – 20, c – 40 and d – 50) mol % Yb^{3+} concentration. There is hardly any change in the size of the nanoparticles observed, indicating that the change in the UCL intensity of CS and CSS nanoparticles are not due to the size of the core but rather associated with other parameters like variation of Yb^{3+} concentration in core as well as in outermost shell, shell thickness, etc.

UC comparison of Ca^{2+}-doped and un-doped core/shell/shell UCNPs

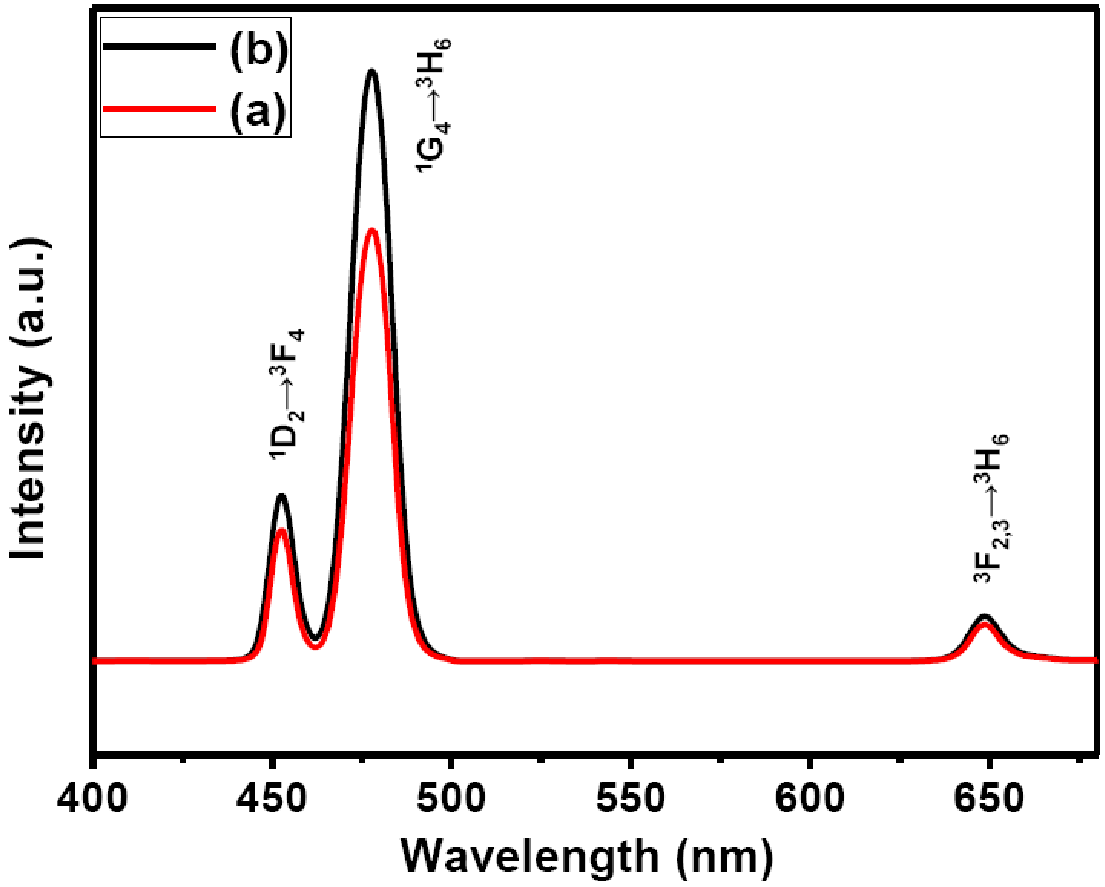

Fig. S11. The UCL spectra of (a) $NaGdF_4$:Tm^{3+}(0.75)/Yb^{3+}(40)/Nd^{3+}(1)@$NaGdF_4$:Nd^{3+}(30) and (b) $NaGdF_4$:Tm^{3+}(0.75)/Yb^{3+}(40)/Nd^{3+}(1)/Ca^{2+}(7)@$NaGdF_4$:Nd^{3+}(30)/Ca^{2+}(7) core/shell UCNPs. Ca^{2+} -dopants modified the lattice defects and concurrently enhanced the energy transfer efficiency. All samples were dispersed in cyclohexane.

TEM images of core/shell/shell UCNPs with different Er^{3+} concentration in the outermost shell

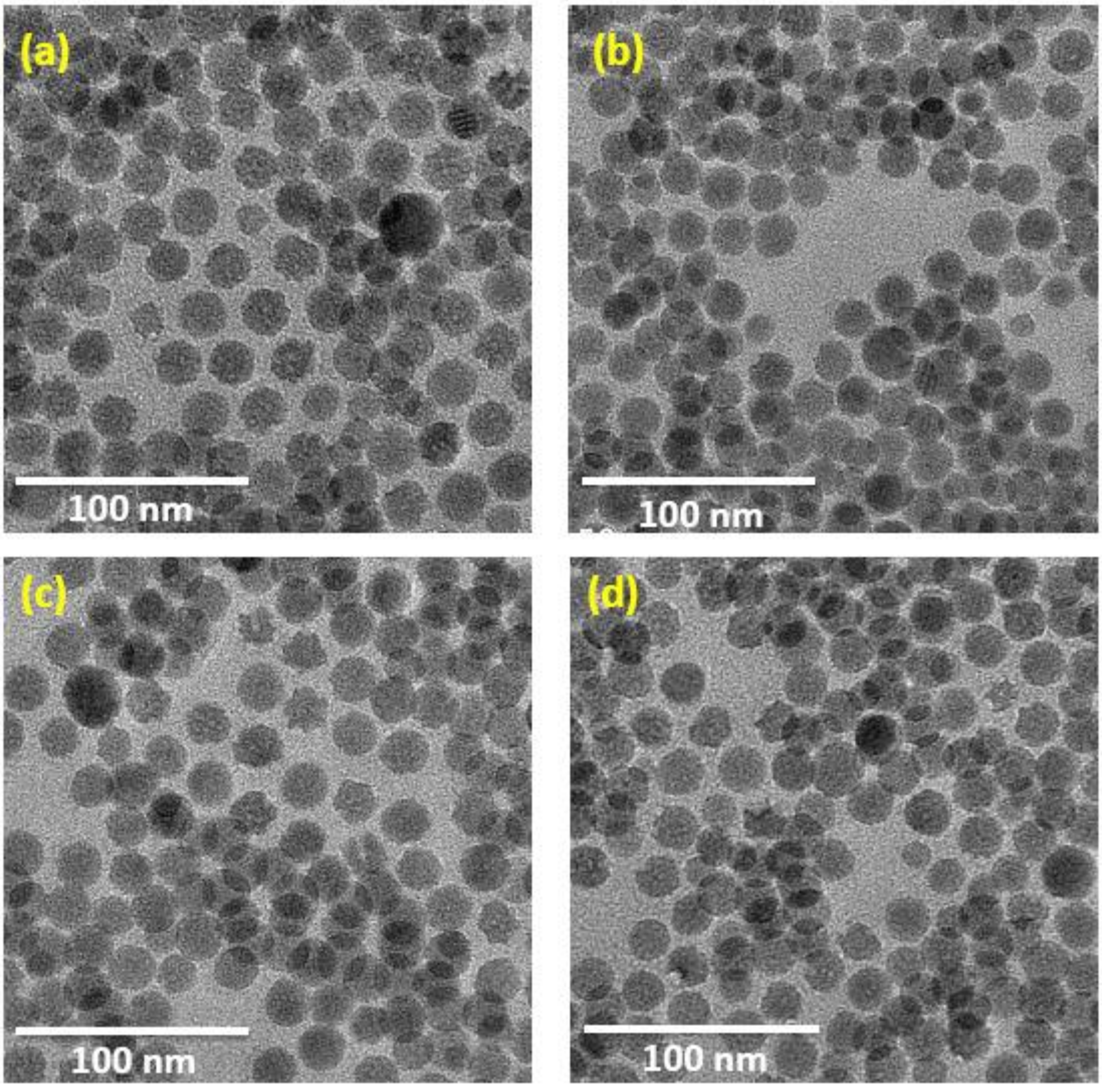

Fig. S12. TEM images of CSS (Er^{3+} = 1, 2, 3 and 7 mol %) UCNPs [(a) = 1 %, (b) = 2 %, (c) = 3 % and (d) = 7 %]. It can be seen that all the obtained core/shell/shell UCNPs have a uniform spherical shape with an average diameter ~19 nm.

Histogram of core/shell/shell UCNPs with different Er^{3+} concentration in the outermost shell

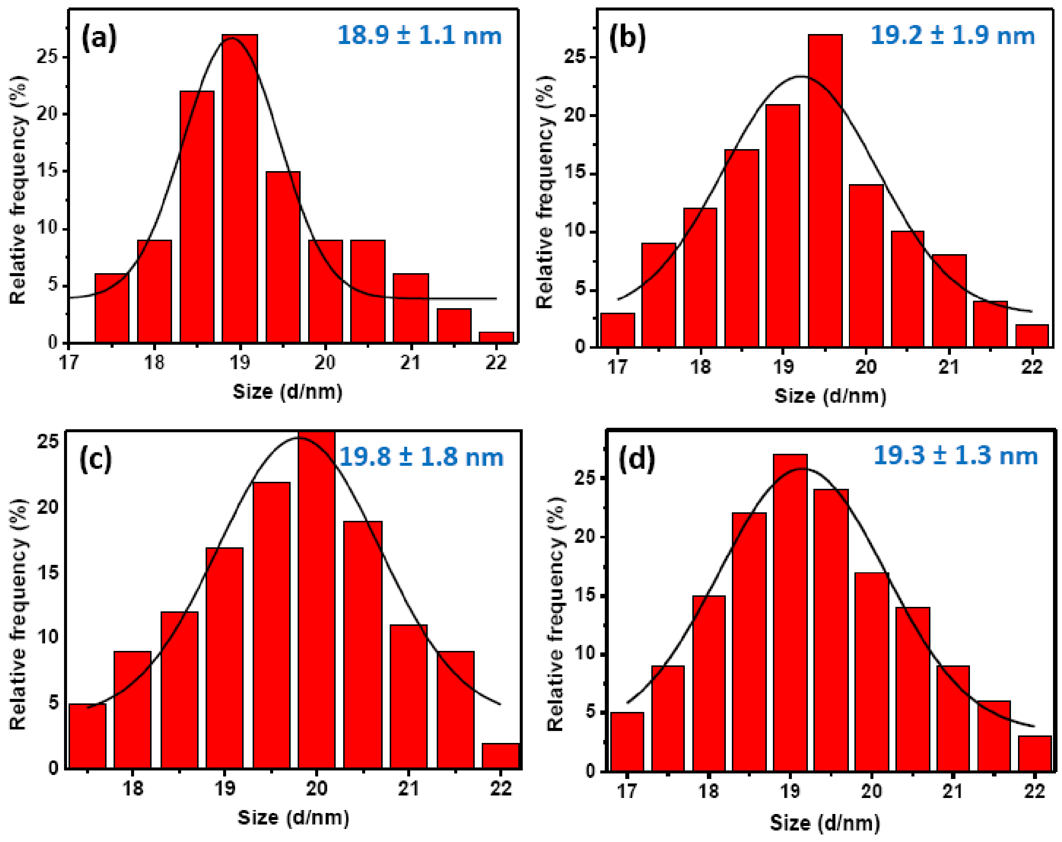

Fig. S13. Size distribution of CSS (Er^{3+} = 1, 2, 3 and 7 mol %) UCNPs [(a) = 1 %, (b) = 2 %, (c) = 3 % and (d) = 7 %]. Statistical analysis was performed on 100 particles in each of the two batches, recurring the respective size distributions of the corresponding nanoparticles. Average diameter (d/nm) of the core/shell/shell UCNPs is shown with significant error.

UCL Comparison of Tm^{3+}-doped, Er^{3+}-doped and Tm^{3+}/Er^{3+} co-doped core/shell/shell UCNPs

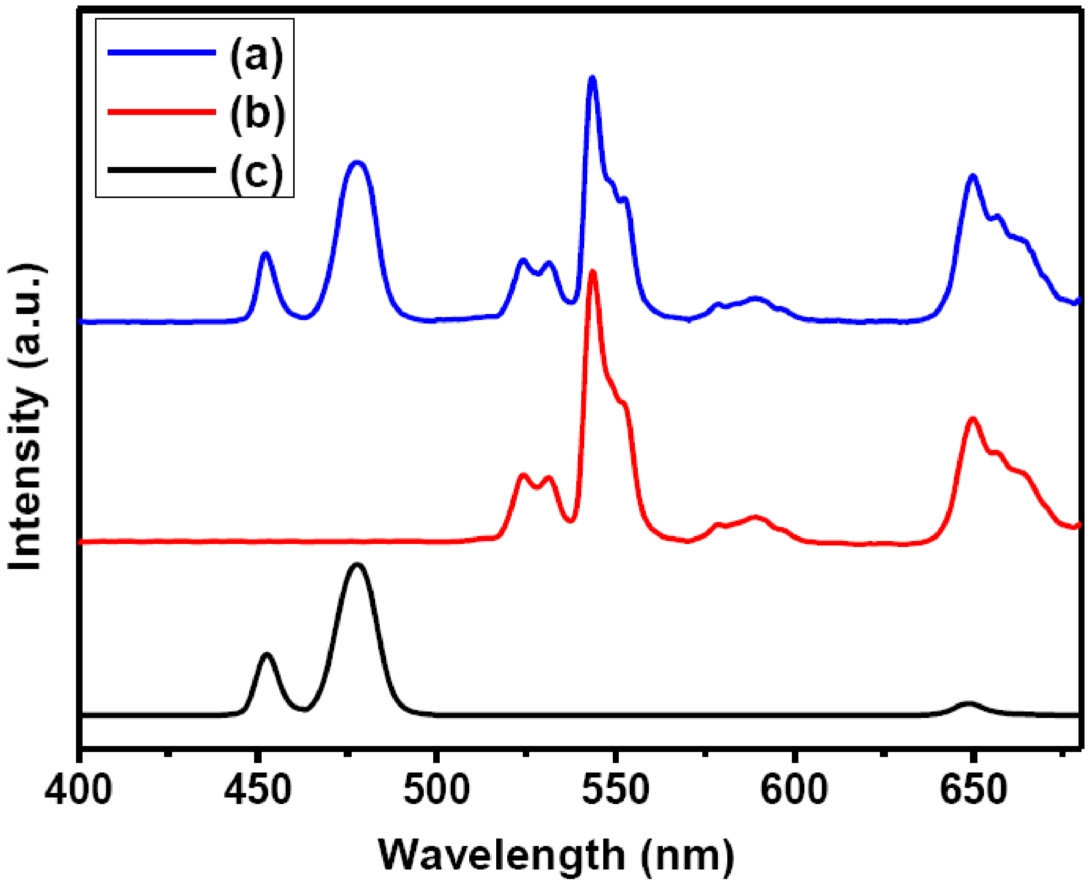

Figure S14. UCL spectra of (a) $NaGdF_4$:Tm^{3+}(0.75)/Yb^{3+}(40)/Nd^{3+}(1)/Ca^{2+}(7)@$NaGdF_4$:Nd^{3+}(30)/Ca^{2+}(7)@$NaGdF_4$:Yb^{3+}(40)/Nd^{3+}(1)/Ca^{2+}(7)/Er^{3+}(5), (b) $NaGdF_4$:Yb^{3+}(40)/Nd^{3+}(1)/Ca^{2+}(7)@$NaGdF_4$:Nd^{3+}(30)/Ca^{2+}(7)@$NaGdF_4$:Yb^{3+}(40)/Nd^{3+}(1)/Ca^{2+}(7)/Er^{3+}(5) and (c) $NaGdF_4$:Tm^{3+}(0.75)/Yb^{3+}(40)/Nd^{3+}(1)/Ca^{2+}(7)@$NaGdF_4$:Nd^{3+}(30)/Ca^{2+}(7)@$NaGdF_4$:Yb^{3+}(40)/Nd^{3+}(1)/Ca^{2+}(7) core/shell/shell UCNPs in cyclohexane.

PL emission spectra of core/shell/shell nanoparticles using direct excitation of Tm^{3+} ion and Er^{3+} ion

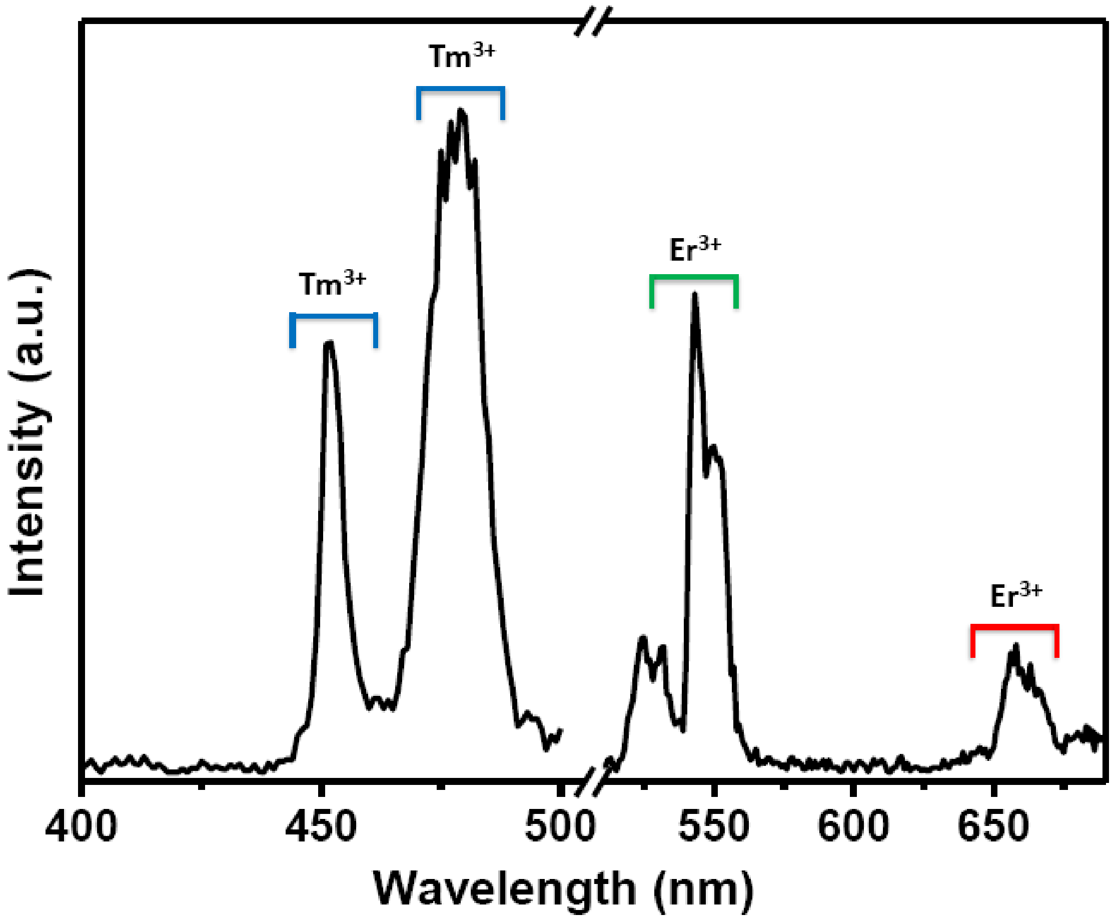

Fig. S15. PL emission spectra of CSS (5 mol % Er^{3+}) [$NaGdF_4$:Tm^{3+}(0.75)/Yb^{3+}(40)/Nd^{3+}(1)/Ca^{2+}(7)@$NaGdF_4$:Nd^{3+}(30)/Ca^{2+}(7)@$NaGdF_4$:Yb^{3+}(40)/Nd^{3+}(1)/Ca^{2+}(7)/Er^{3+}(5)] nanoparticles using direct excitation of Tm^{3+} ions (λ_{exi} = 357 nm) and Er^{3+} ions (λ_{exi} = 488 nm). The integrated PL emission intensity of Tm^{3+} ion (λ_{exi} = 357 nm) was slightly higher (1.3 times) than that of Er^{3+} ion (λ_{exi} = 488 nm). All measurements were carried out under identical condition.

Calculation of photons involved in the white UCL

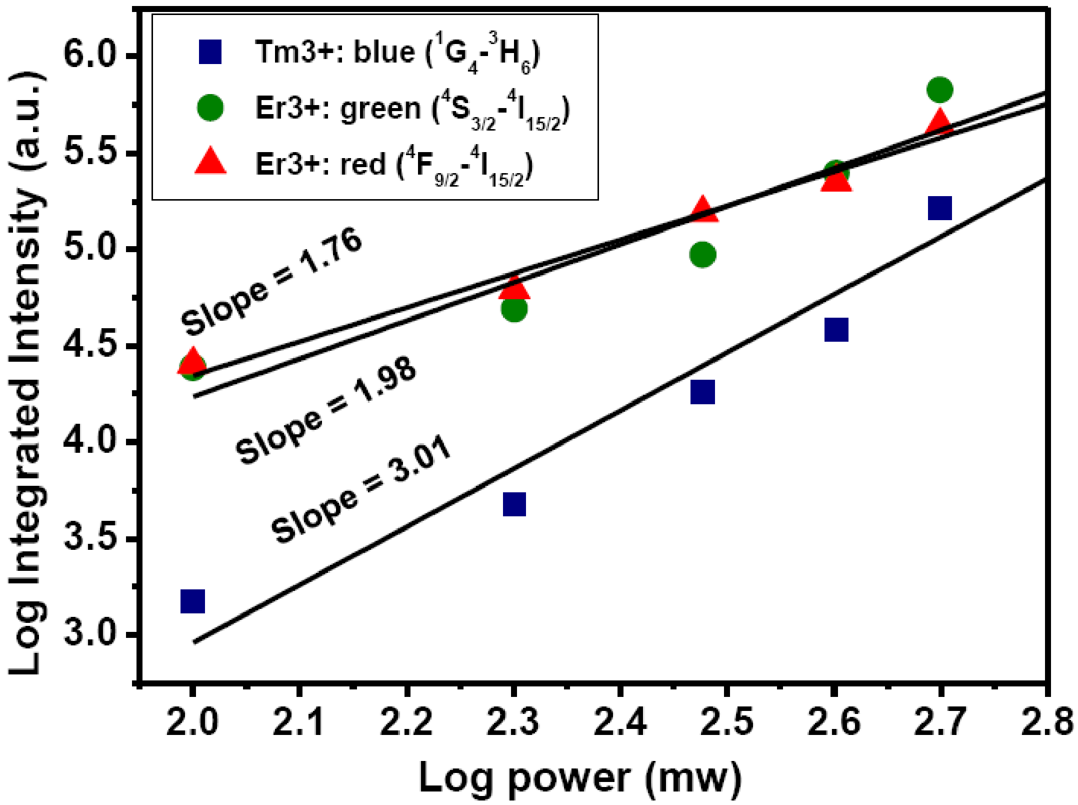

Figure S16. The dependence of the intensities of UCL at 477 nm (Tm^{3+}), 544 nm (Er^{3+}) and 650 nm (Er^{3+}) on the excitation power in colloidal CSS (5 mol % Er^{3+}) [$NaGdF_4$:Tm^{3+}(0.75)/Yb^{3+}(40)/Nd^{3+}(1)/Ca^{2+}(7)@$NaGdF_4$:Nd^{3+}(30)/Ca^{2+}(7)@$NaGdF_4$:Yb^{3+}(40)/Nd^{3+}(1)/Ca^{2+}(7)/Er^{3+}(5)] UCNPs. The number of photon process can be derived from the relation, $I_l \propto P^n$, where I_l is the integrated luminescence intensity and P is the pump laser power and n is the number of the laser photons required to populate the upper emitting state. The value of n can be obtained from the slope of the fitting line in the plot of log I_l versus log p. A slope value of 3.01, 1.98, and 1.76 are observed for the blue emission (Tm^{3+}), green emission (Er^{3+}) and red emission (Er^{3+}), illustrating an involvement of three, two and two-photon process in the generation of UCL at 477 nm, 544 nm and 650 nm, respectively.
